# Impact of Omicron infection on childhood health: the Beijing long-COVID study

**DOI:** 10.3389/fpubh.2025.1377745

**Published:** 2025-02-19

**Authors:** Bo Zhou, Qi Xu, Shaoli Li, Jianhong Wang, Junting Liu, Ting Zhang, Xia Qu, Xi Wang, Lili Zhang, Xiaoli Liu, Jialu Gu, Lijun Zhou, Fangfang Chen, Xinnan Zong, Wenquan Niu, Lin Wang

**Affiliations:** ^1^Child Healthcare Center, Children’s Hospital, Capital Institute of Pediatrics, Beijing, China; ^2^Child Health Big Data Research Center, Capital Institute of Pediatrics, Beijing, China; ^3^Laboratory of Child Development and Nutriomics, Capital Institute of Pediatrics, Beijing, China; ^4^Department of Epidemiology, Capital Institute of Pediatrics, Beijing, China; ^5^Department of Growth and Development, Capital Institute of Pediatrics, Beijing, China; ^6^Center for Evidence-Based Medicine, Capital Institute of Pediatrics, Beijing, China

**Keywords:** Omicron infection, long Covid, children, Symptom Burden Questionnaire, prospective cohort

## Abstract

**Purpose:**

The aim of this prospective study was to assess the dynamic changes of persisting symptoms among children aged 6–18 years during 1–2 months after the Omicron infection based on the modified SBQ-LC in the Tongzhou cohort, Beijing.

**Methods:**

This study includes 4 serial surveys performed within January 7–9, January 14–16, January 21–23, and February 12–14 in 2023, respectively. The prediction of age and survey for eight domains in the Rasch 0–100 linear score was undertaken by generalized additive mixed model.

**Results:**

Total 1,536 children (median age: 13 years, boys: 49.9%) had completed questionnaires across 4 surveys. Information on 51 symptoms was collected, with each scored on a 4-point rating scale. Generally, the distribution of age with all domains followed the N-shaped geometry, and that of survey followed the inverse J-shaped geometry. The Rasch linear score hit the lowest level among children aged 6–8 years, and reached the peak among children aged 12–13 years. The scores of all domains sharply declined from the first to the third survey, and remained stable between the third and the fourth survey. At the fourth survey, 95.05 and 51.37% children still had one or more problems relating to breathing and mental health, respectively, and the percentage of rest six domains was reduced to less than 20%.

**Conclusion:**

Our findings indicate the multifaceted impact of Omicron infection on childhood health, especially among children aged 12–13 years. Moreover, breathing and mental health related problems still persisted during 1-to-2-month Omicron infection period.

## Introduction

Coronavirus disease 2019 (COVID-19), caused by severe acute respiratory syndrome coronavirus 2 (SARS-CoV-2), has made a serious public health threat worldwide spreading to over 200 countries globally and infecting millions of people of all ages. Since firstly reported on November 9, 2021, the highly-contagious Omicron variant has swiftly swept around the world (1). In China, the fight against Omicron has lasted nearly a whole year, and on December 8, 2022, the epidemic prevention and control policy was officially optimized by releasing the zero-COVID approaches and lifting mandatory quarantine measures. As expected, the sudden arrival of Omicron has fueled a surge in coronavirus-infected cases across China, affecting all age groups, including children (2, 3). In spite of stronger transmission and immune escape abilities, Omicron infection is associated with shorter recovery time and lower fatality rate, and it seems that Omicron variants affect a large proportion of the younger population with the appearance of clinical manifestations similar to adults (4–6). The majority of children have fully recovered within 1 month following Omicron infection, yet a fraction of them still experienced one or more unexplained persistent residual or new-onset symptoms afterwards. These symptoms, collectively termed as long COVID or post-COVID sequelae, have constituted a major public health issue in the post-pandemic era (7). On average, one in four children experienced long COVID, which can more or less cause damage to many organ systems, possibly *via* fatigue, post-exertional dyspnea, headache, or sleep disorders (8). For example, Hassan and colleagues have conducted an excellent meta-analysis on the prevalence of mental health problems among children with post-COVID sequelae, by demonstrating that the higher prevalence of anxiety, depression and appetite problems among infected children than those uninfected might be attributable to long COVID (9). Moreover, a national cross-sectional study from Israel showed that symptoms including headache, weakness, fatigue and abdominal pain were more common among children with COVID infection than those without (10). Thus far, evidence on post-Omicron symptoms in children is primarily based on cross-sectional observations or case series, and a dynamic continuum for these symptoms are lacking. What’s more, a literature search has failed to reveal any evidence from Chinese children on this subject.

To fill this gap in knowledge and yield more reference for following investigations, we adopted the modified Symptom Burden Questionnaire for long COVID (SBQ-LC) (11) and prospectively surveyed children and adolescents 6–18 years of age in the Tongzhou district, Beijing from January 7 to February 14, 2023 at four serial intervals, aiming to assess the dynamic changes of persisting symptoms after Omicron waves from eight domains.

## Methods

### Study design

This is a prospective study involving four cross-sectional surveys performed on January 7–9, January 14–16, January 21–23 and February 12–14, respectively, in parallel to post-Omicron 2–3 weeks, 3–4 weeks, 4–5 weeks, and 8–9 weeks. The conduct of this study was reviewed and approved by the Ethics Committee at the Capital Institute of Pediatrics (Beijing, China), and informed consent was obtained from the parents or major supervisors of all study participants.

### Study participants

The data for this study were obtained from the Beijing Children and Adolescents Health Cohort conducted from 2022 to 2023, which was a prospective cohort study design established in Tongzhou district of Beijing. The cohort consisted of three primary, three junior and three high schools. Two classes of each grade in each school were randomly selected for cluster sampling survey. All school-aged children 6–18 years were surveyed using online questionnaires implemented on the Wenjuanxing platform.^1^ Questionnaires, circulated four times following the Omicron waves, were filled out by the parents or major supervisors of children involved. Children were excluded from this study if they were clinically diagnosed to have respiratory, digestive, cardiovascular, psychological and behavioral diseases prior to COVID-19 infection.

**Figure 1:**
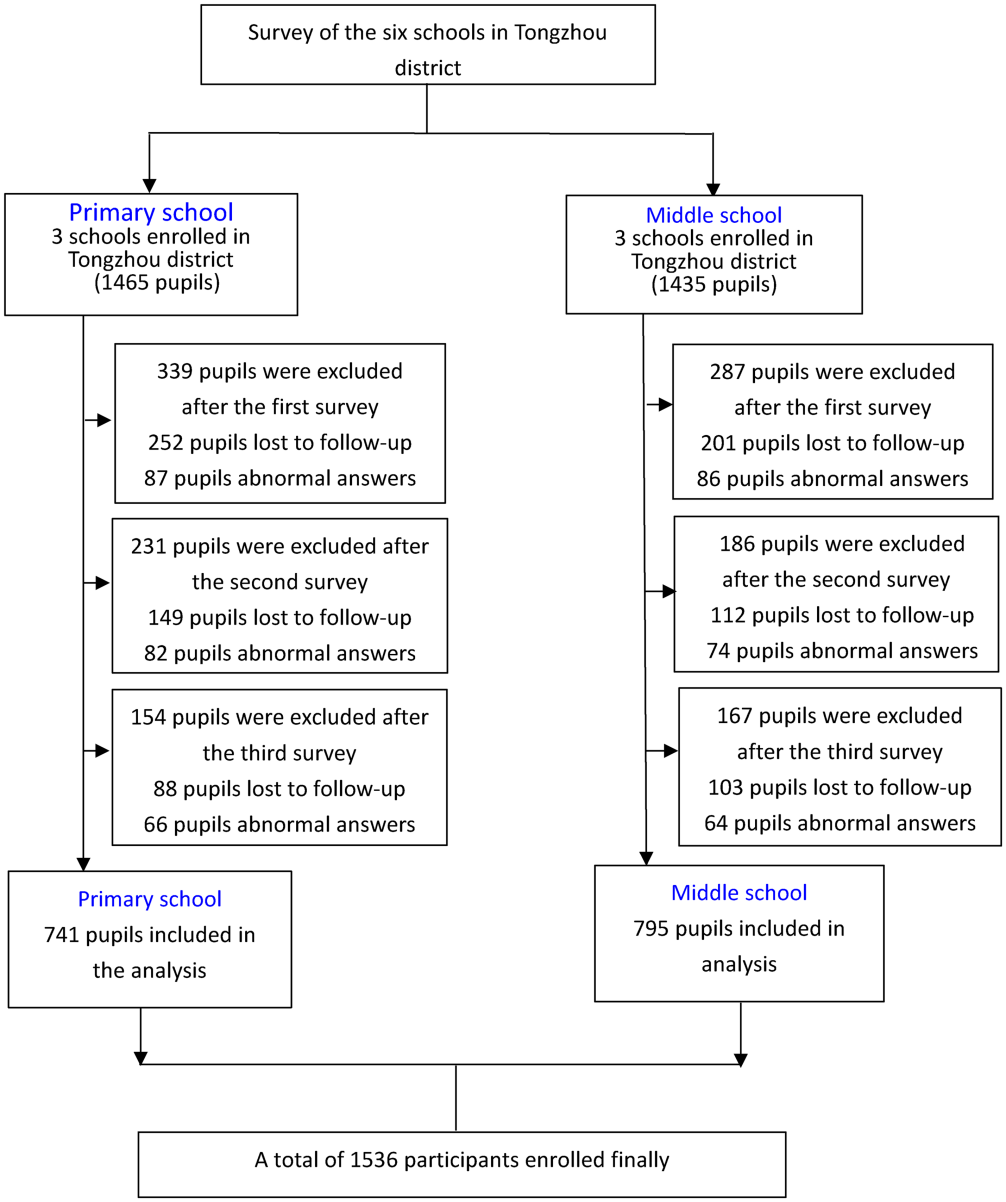
CONSORT diagram.

Initially, 2,900 children were surveyed, and due to incomplete information across four surveys, only 1,536 children formed the present analytical cohort. The study profile was described in Figure 1.

### Questionnaire

The questionnaire used in this study referred to the SBQ-LC version 1.0 (11), a comprehensive patient reported outcome instrument developed using modern psychometric methods. Considering that the SBQ-LC is primarily designed for adults, only domains suitable for children were selected. Specially, the original SBQ-LC was constructed based on 123 symptoms from 16 domains, and the modified SBQ-LC for children on 51 symptoms from 8 domains, that is, breathing (*n* = 7), pain (*n* = 4), circulation (*n* = 5), fatigue (*n* = 4), memory and thinking and communication (*n* = 10), movement (*n* = 3), muscles and joints (*n* = 9) and mental health (*n* = 9). Symptoms referred to the best description of experience over the last 7 days, and were scored on 4-point scales according to symptom severity, with 0 being none, 1 being mild, 2 being moderate and 3 being severe. The scale raw score of each domain was conversed to the Rasch 0–100 linear score, with higher scale scores denoting greater symptom burden for each domain. The 51 symptoms were translated into Chinese language with maintained integrity.

### Quality control

To avoid duplicated reports, the same device (smart phone) can be used only once to complete questionnaires at each survey. No personal information including names and identification number was covered in the questionnaire to ensure total anonymity and honest responses. In addition, to ensure information integrity, submitting questionnaires cannot be performed until the competition of all items. The questionnaire settings provided control over the questionnaires and prevented respondents from random selection.

### Statistical analyses

Continuous data are expressed as mean (standard deviation) in the case of normal distributions or as median (interquartile range) in the case of skewed distributions. Categorical data are expressed as count (percent). Difference of continuous data between four surveys was tested by using the Analysis of Variance (ANOVA), and that of categorical data using the χ^2^-test. The joint distribution of age and sex was displayed by pyramid plot. Correlation of scale scores of eight domains was evaluated by the Spearman correlation coefficients upon stratified by surveys and displayed separately using heat maps. The prediction of age and survey for eight domains in the Rasch 0–100 linear score was undertaken by means of generalized additive mixed model, with knot values specified as 4 and using the Gaussian function with identity link. The age and survey were explored as smoothed factors, and sex as non-smoothed factor. Two-dimension (age or survey with each domain in the modified SBQ-LC) and three-dimension (age, survey and each domain) were depicted to inspect underlying nonlinear changes. The distribution of symptom combination within each domain was inspected using the Venn plot for four surveys. Statistical analyses and data representation were completed using the R programming language version 4.3.0 implemented under the Integrated Development Environment RStudio version 2023.03.1.

## Results

### Characteristics of study participants

Of all study participants, 1,536 children had completed information across 4 surveys, and their basic characteristics and symptom distributions are shown in Table 1. The median age of 1,536 study children was 13 years, and 49.9% of them were boys. In total, 51 symptoms were surveyed, and each was scored on a 4-point rating scale.

**Table 1:** Symptom distributions of children and adolescents across 4 surveys.

Characteristics		The 1st survey	The 2nd survey	The 3rd survey	The 4th survey	*p*
Sample size		1,536	1,536	1,536	1,536	
age (median [IQR])		13.00 [9.00, 15.00]				NA
Boys (%)		767 (49.9)				NA
Physical mental exhaustion (%)	None	555 (36.1)	1,043 (67.9)	1,295 (84.3)	1,340 (87.2)	<0.001
	Mild	459 (29.9)	280 (18.2)	173 (11.3)	154 (10.0)	
	Moderate	421 (27.4)	161 (10.5)	54 (3.5)	31 (2.0)	
	Severe	101 (6.6)	52 (3.4)	14 (0.9)	11 (0.7)	
Low energy (%)	None	524 (34.1)	1,033 (67.3)	1,301 (84.7)	1,342 (87.4)	<0.001
	Mild	459 (29.9)	296 (19.3)	168 (10.9)	149 (9.7)	
	Moderate	438 (28.5)	165 (10.7)	53 (3.5)	35 (2.3)	
	Severe	115 (7.5)	42 (2.7)	14 (0.9)	10 (0.7)	
Tiredness (%)	None	613 (39.9)	1,077 (70.1)	1,316 (85.7)	1,338 (87.1)	<0.001
	Mild	473 (30.8)	267 (17.4)	161 (10.5)	150 (9.8)	
	Moderate	356 (23.2)	150 (9.8)	45 (2.9)	35 (2.3)	
	Severe	94 (6.1)	42 (2.7)	14 (0.9)	13 (0.8)	
Worsening symptoms after activity (%)	None	610 (39.7)	1,071 (69.7)	1,293 (84.2)	1,345 (87.6)	<0.001
	Mild	476 (31.0)	287 (18.7)	181 (11.8)	147 (9.6)	
	Moderate	344 (22.4)	134 (8.7)	47 (3.1)	31 (2.0)	
	Severe	106 (6.9)	44 (2.9)	15 (1.0)	13 (0.8)	
Shortness of breathing sitting (%)	None	1,028 (66.9)	1,249 (81.3)	1,395 (90.8)	1,425 (92.8)	<0.001
	Mild	358 (23.3)	202 (13.2)	109 (7.1)	78 (5.1)	
	Moderate	114 (7.4)	58 (3.8)	23 (1.5)	23 (1.5)	
	Severe	36 (2.3)	27 (1.8)	9 (0.6)	10 (0.7)	
Shortness of breathing staring (%)	None	1,036 (67.4)	1,215 (79.1)	1,368 (89.1)	1,397 (91.0)	<0.001
	Mild	337 (21.9)	227 (14.8)	134 (8.7)	106 (6.9)	
	Moderate	122 (7.9)	67 (4.4)	25 (1.6)	25 (1.6)	
	Severe	41 (2.7)	27 (1.8)	9 (0.6)	8 (0.5)	
Shortness of breathing lying (%)	None	1,127 (73.4)	1,278 (83.2)	1,409 (91.7)	1,429 (93.0)	<0.001
	Mild	282 (18.4)	177 (11.5)	101 (6.6)	77 (5.0)	
	Moderate	99 (6.4)	55 (3.6)	17 (1.1)	21 (1.4)	
	Severe	28 (1.8)	26 (1.7)	9 (0.6)	9 (0.6)	
Chest tightness (%)	None	1,134 (73.8)	1,279 (83.3)	1,415 (92.1)	1,441 (93.8)	<0.001
	Mild	265 (17.3)	172 (11.2)	97 (6.3)	67 (4.4)	
	Moderate	104 (6.8)	62 (4.0)	15 (1.0)	20 (1.3)	
	Severe	33 (2.1)	23 (1.5)	9 (0.6)	8 (0.5)	
Wheezing noisy breathing (%)	None	964 (62.8)	1,220 (79.4)	1,394 (90.8)	1,427 (92.9)	<0.001
	Mild	355 (23.1)	212 (13.8)	110 (7.2)	74 (4.8)	
	Moderate	156 (10.2)	77 (5.0)	23 (1.5)	26 (1.7)	
	Severe	61 (4.0)	27 (1.8)	9 (0.6)	9 (0.6)	
Wakeup short of breath (%)	None	1,298 (84.5)	1,405 (91.5)	1,460 (95.1)	1,458 (94.9)	<0.001
	Mild	238 (15.5)	131 (8.5)	76 (4.9)	78 (5.1)	
Breathing faster than usual (%)	None	360 (23.4)	271 (17.6)	166 (10.8)	166 (10.8)	<0.001
	Mild	1,176 (76.6)	1,265 (82.4)	1,370 (89.2)	1,370 (89.2)	
Difficulty remembering (%)	None	1,536 (100.0)	1,536 (100.0)	1,536 (100.0)	1,536 (100.0)	NA
Memory loss (%)	None	1,094 (71.2)	1,186 (77.2)	1,317 (85.7)	1,339 (87.2)	<0.001
	Mild	337 (21.9)	271 (17.6)	177 (11.5)	158 (10.3)	
	Moderate	83 (5.4)	53 (3.5)	28 (1.8)	25 (1.6)	
	Severe	22 (1.4)	26 (1.7)	14 (0.9)	14 (0.9)	
Brain frog (%)	None	1,136 (74.0)	1,213 (79.0)	1,341 (87.3)	1,379 (89.8)	<0.001
	Mild	299 (19.5)	244 (15.9)	157 (10.2)	120 (7.8)	
	Moderate	77 (5.0)	51 (3.3)	24 (1.6)	23 (1.5)	
	Severe	24 (1.6)	28 (1.8)	14 (0.9)	14 (0.9)	
Difficulty planning (%)	None	1,059 (68.9)	1,200 (78.1)	1,356 (88.3)	1,380 (89.8)	<0.001
	Mild	342 (22.3)	236 (15.4)	142 (9.2)	120 (7.8)	
	Moderate	103 (6.7)	72 (4.7)	23 (1.5)	25 (1.6)	
	Severe	32 (2.1)	28 (1.8)	15 (1.0)	11 (0.7)	
Confusion happening (%)	None	1,270 (82.7)	1,308 (85.2)	1,403 (91.3)	1,424 (92.7)	<0.001
	Mild	194 (12.6)	160 (10.4)	110 (7.2)	82 (5.3)	
	Moderate	51 (3.3)	42 (2.7)	13 (0.8)	18 (1.2)	
	Severe	21 (1.4)	26 (1.7)	10 (0.7)	12 (0.8)	
Difficulty concentrating (%)	None	972 (63.3)	1,188 (77.3)	1,325 (86.3)	1,365 (88.9)	<0.001
	Mild	411 (26.8)	251 (16.3)	173 (11.3)	137 (8.9)	
	Moderate	121 (7.9)	71 (4.6)	26 (1.7)	22 (1.4)	
	Severe	32 (2.1)	26 (1.7)	12 (0.8)	12 (0.8)	
Word finding difficulty (%)	None	1,249 (81.3)	1,310 (85.3)	1,405 (91.5)	1,430 (93.1)	<0.001
	Mild	219 (14.3)	163 (10.6)	105 (6.8)	80 (5.2)	
	Moderate	52 (3.4)	43 (2.8)	18 (1.2)	15 (1.0)	
	Severe	16 (1.0)	20 (1.3)	8 (0.5)	11 (0.7)	
Understanding difficulty (%)	None	1,315 (85.6)	1,344 (87.5)	1,410 (91.8)	1,428 (93.0)	<0.001
	Mild	164 (10.7)	147 (9.6)	101 (6.6)	81 (5.3)	
	Moderate	42 (2.7)	29 (1.9)	16 (1.0)	18 (1.2)	
	Severe	15 (1.0)	16 (1.0)	9 (0.6)	9 (0.6)	
Slurred speech (%)	None	1,299 (84.6)	1,322 (86.1)	1,417 (92.3)	1,434 (93.4)	<0.001
	Mild	178 (11.6)	159 (10.4)	98 (6.4)	74 (4.8)	
	Moderate	46 (3.0)	36 (2.3)	14 (0.9)	19 (1.2)	
	Severe	13 (0.8)	19 (1.2)	7 (0.5)	9 (0.6)	
Reading difficulty (%)	None	1,301 (84.7)	1,330 (86.6)	1,424 (92.7)	1,429 (93.0)	<0.001
	Mild	175 (11.4)	150 (9.8)	85 (5.5)	83 (5.4)	
	Moderate	43 (2.8)	36 (2.3)	20 (1.3)	14 (0.9)	
	Severe	17 (1.1)	20 (1.3)	7 (0.5)	10 (0.7)	
Tremor (%)	None	1,317 (85.7)	1,348 (87.8)	1,432 (93.2)	1,448 (94.3)	<0.001
	Mild	169 (11.0)	135 (8.8)	77 (5.0)	65 (4.2)	
	Moderate	38 (2.5)	36 (2.3)	15 (1.0)	14 (0.9)	
	Severe	12 (0.8)	17 (1.1)	12 (0.8)	9 (0.6)	
Balance difficulty (%)	None	1,260 (82.0)	1,313 (85.5)	1,414 (92.1)	1,422 (92.6)	<0.001
	Mild	199 (13.0)	161 (10.5)	95 (6.2)	89 (5.8)	
	Moderate	56 (3.6)	44 (2.9)	16 (1.0)	18 (1.2)	
	Severe	21 (1.4)	18 (1.2)	11 (0.7)	7 (0.5)	
Movement difficulty (%)	None	1,255 (81.7)	1,315 (85.6)	1,421 (92.5)	1,425 (92.8)	<0.001
	Mild	216 (14.1)	159 (10.4)	90 (5.9)	91 (5.9)	
	Moderate	49 (3.2)	43 (2.8)	16 (1.0)	12 (0.8)	
	Severe	16 (1.0)	19 (1.2)	9 (0.6)	8 (0.5)	
Feeling faint (%)	None	1,058 (68.9)	1,224 (79.7)	1,355 (88.2)	1,394 (90.8)	<0.001
	Mild	307 (20.0)	193 (12.6)	138 (9.0)	95 (6.2)	
	Moderate	124 (8.1)	90 (5.9)	31 (2.0)	33 (2.1)	
	Severe	47 (3.1)	29 (1.9)	12 (0.8)	14 (0.9)	
Palpitations (%)	None	1,335 (86.9)	1,369 (89.1)	1,431 (93.2)	1,440 (93.8)	<0.001
	Mild	152 (9.9)	112 (7.3)	82 (5.3)	66 (4.3)	
	Moderate	36 (2.3)	38 (2.5)	13 (0.8)	22 (1.4)	
	Severe	13 (0.8)	17 (1.1)	10 (0.7)	8 (0.5)	
Dizziness standing (%)	None	1,058 (68.9)	1,224 (79.7)	1,355 (88.2)	1,394 (90.8)	<0.001
	Mild	307 (20.0)	193 (12.6)	138 (9.0)	95 (6.2)	
	Moderate	124 (8.1)	90 (5.9)	31 (2.0)	33 (2.1)	
	Severe	47 (3.1)	29 (1.9)	12 (0.8)	14 (0.9)	
Swelling legs and feet (%)	None	1,424 (92.7)	1,413 (92.0)	1,458 (94.9)	1,469 (95.6)	0.002
	Mild	83 (5.4)	85 (5.5)	57 (3.7)	44 (2.9)	
	Moderate	21 (1.4)	26 (1.7)	14 (0.9)	14 (0.9)	
	Severe	8 (0.5)	12 (0.8)	7 (0.5)	9 (0.6)	
Hands and feet colder than usual (%)	None	1,115 (72.6)	1,228 (79.9)	1,367 (89.0)	1,394 (90.8)	<0.001
	Mild	261 (17.0)	198 (12.9)	120 (7.8)	91 (5.9)	
	Moderate	114 (7.4)	81 (5.3)	37 (2.4)	38 (2.5)	
	Severe	46 (3.0)	29 (1.9)	12 (0.8)	13 (0.8)	
Chest pain (%)	None	1,348 (87.8)	1,384 (90.1)	1,452 (94.5)	1,462 (95.2)	<0.001
	Mild	134 (8.7)	111 (7.2)	64 (4.2)	48 (3.1)	
	Moderate	43 (2.8)	27 (1.8)	11 (0.7)	17 (1.1)	
	Severe	11 (0.7)	14 (0.9)	9 (0.6)	9 (0.6)	
Pain on breathing (%)	None	1,337 (87.0)	1,379 (89.8)	1,457 (94.9)	1,463 (95.2)	<0.001
	Mild	126 (8.2)	110 (7.2)	59 (3.8)	53 (3.5)	
	Moderate	53 (3.5)	31 (2.0)	11 (0.7)	10 (0.7)	
	Severe	20 (1.3)	16 (1.0)	9 (0.6)	10 (0.7)	
Shooting stabbing pain (%)	None	1,295 (84.3)	1,368 (89.1)	1,436 (93.5)	1,450 (94.4)	<0.001
	Mild	151 (9.8)	114 (7.4)	77 (5.0)	59 (3.8)	
	Moderate	72 (4.7)	38 (2.5)	13 (0.8)	16 (1.0)	
	Severe	18 (1.2)	16 (1.0)	10 (0.7)	11 (0.7)	
Aching burning pain (%)	None	1,341 (87.3)	1,385 (90.2)	1,458 (94.9)	1,463 (95.2)	<0.001
	Mild	134 (8.7)	98 (6.4)	55 (3.6)	51 (3.3)	
	Moderate	47 (3.1)	34 (2.2)	15 (1.0)	15 (1.0)	
	Severe	14 (0.9)	19 (1.2)	8 (0.5)	7 (0.5)	
Muscle pain (%)	None	1,053 (68.6)	1,265 (82.4)	1,407 (91.6)	1,420 (92.4)	<0.001
	Mild	304 (19.8)	183 (11.9)	96 (6.2)	80 (5.2)	
	Moderate	140 (9.1)	63 (4.1)	22 (1.4)	27 (1.8)	
	Severe	39 (2.5)	25 (1.6)	11 (0.7)	9 (0.6)	
Muscle weakness (%)	None	1,005 (65.4)	1,227 (79.9)	1,402 (91.3)	1,404 (91.4)	<0.001
	Mild	337 (21.9)	204 (13.3)	100 (6.5)	94 (6.1)	
	Moderate	153 (10.0)	76 (4.9)	22 (1.4)	29 (1.9)	
	Severe	41 (2.7)	29 (1.9)	12 (0.8)	9 (0.6)	
Muscle stiffness (%)	None	1,297 (84.4)	1,368 (89.1)	1,450 (94.4)	1,453 (94.6)	<0.001
	Mild	161 (10.5)	115 (7.5)	66 (4.3)	57 (3.7)	
	Moderate	53 (3.5)	35 (2.3)	13 (0.8)	18 (1.2)	
	Severe	25 (1.6)	18 (1.2)	7 (0.5)	8 (0.5)	
Joint pain (%)	None	1,179 (76.8)	1,316 (85.7)	1,426 (92.8)	1,441 (93.8)	<0.001
	Mild	222 (14.5)	149 (9.7)	82 (5.3)	65 (4.2)	
	Moderate	100 (6.5)	48 (3.1)	15 (1.0)	19 (1.2)	
	Severe	35 (2.3)	23 (1.5)	13 (0.8)	11 (0.7)	
Joint swelling (%)	None	1,409 (91.7)	1,420 (92.4)	1,457 (94.9)	1,467 (95.5)	0.001
	Mild	95 (6.2)	77 (5.0)	57 (3.7)	48 (3.1)	
	Moderate	22 (1.4)	25 (1.6)	14 (0.9)	14 (0.9)	
	Severe	10 (0.7)	14 (0.9)	8 (0.5)	7 (0.5)	
Joint stiffness (%)	None	1,402 (91.3)	1,417 (92.3)	1,458 (94.9)	1,463 (95.2)	<0.001
	Mild	95 (6.2)	78 (5.1)	60 (3.9)	51 (3.3)	
	Moderate	26 (1.7)	28 (1.8)	11 (0.7)	14 (0.9)	
	Severe	13 (0.8)	13 (0.8)	7 (0.5)	8 (0.5)	
Muscle twitching (%)	None	1,415 (92.1)	1,420 (92.4)	1,454 (94.7)	1,468 (95.6)	0.003
	Mild	92 (6.0)	80 (5.2)	60 (3.9)	51 (3.3)	
	Moderate	19 (1.2)	24 (1.6)	12 (0.8)	11 (0.7)	
	Severe	10 (0.7)	12 (0.8)	10 (0.7)	6 (0.4)	
Muscle cramping (%)	None	1,422 (92.6)	1,426 (92.8)	1,459 (95.0)	1,462 (95.2)	0.034
	Mild	88 (5.7)	77 (5.0)	59 (3.8)	54 (3.5)	
	Moderate	18 (1.2)	21 (1.4)	10 (0.7)	13 (0.8)	
	Severe	8 (0.5)	12 (0.8)	8 (0.5)	7 (0.5)	
Tingling numbness (%)	None	1,332 (86.7)	1,385 (90.2)	1,450 (94.4)	1,457 (94.9)	<0.001
	Mild	148 (9.6)	108 (7.0)	67 (4.4)	58 (3.8)	
	Moderate	37 (2.4)	29 (1.9)	10 (0.7)	12 (0.8)	
	Severe	19 (1.2)	14 (0.9)	9 (0.6)	9 (0.6)	
Lack interest (%)	None	1,199 (78.1)	1,300 (84.6)	1,397 (91.0)	1,396 (90.9)	<0.001
	Mild	246 (16.0)	165 (10.7)	105 (6.8)	104 (6.8)	
	Moderate	63 (4.1)	52 (3.4)	22 (1.4)	22 (1.4)	
	Severe	28 (1.8)	19 (1.2)	12 (0.8)	14 (0.9)	
Feeling anxious (%)	None	1,150 (74.9)	1,266 (82.4)	1,361 (88.6)	1,376 (89.6)	<0.001
	Mild	272 (17.7)	185 (12.0)	130 (8.5)	117 (7.6)	
	Moderate	83 (5.4)	61 (4.0)	31 (2.0)	30 (2.0)	
	Severe	31 (2.0)	24 (1.6)	14 (0.9)	13 (0.8)	
Feeling sad (%)	None	1,312 (85.4)	1,363 (88.7)	1,427 (92.9)	1,430 (93.1)	<0.001
	Mild	158 (10.3)	118 (7.7)	78 (5.1)	73 (4.8)	
	Moderate	46 (3.0)	36 (2.3)	18 (1.2)	24 (1.6)	
	Severe	20 (1.3)	19 (1.2)	13 (0.8)	9 (0.6)	
Thoughts self-harm (%)	None	1,430 (93.1)	1,417 (92.3)	1,454 (94.7)	1,453 (94.6)	0.038
	Mild	79 (5.1)	78 (5.1)	61 (4.0)	62 (4.0)	
	Moderate	22 (1.4)	27 (1.8)	12 (0.8)	14 (0.9)	
	Severe	5 (0.3)	14 (0.9)	9 (0.6)	7 (0.5)	
Mood swings (%)	None	1,138 (74.1)	1,257 (81.8)	1,371 (89.3)	1,394 (90.8)	<0.001
	Mild	286 (18.6)	197 (12.8)	126 (8.2)	104 (6.8)	
	Moderate	88 (5.7)	61 (4.0)	26 (1.7)	25 (1.6)	
	Severe	24 (1.6)	21 (1.4)	13 (0.8)	13 (0.8)	
Change in appetite (%)	None	971 (63.2)	1,201 (78.2)	1,348 (87.8)	1,383 (90.0)	<0.001
	Mild	330 (21.5)	201 (13.1)	133 (8.7)	105 (6.8)	
	Moderate	176 (11.5)	101 (6.6)	36 (2.3)	33 (2.1)	
	Severe	59 (3.8)	33 (2.1)	19 (1.2)	15 (1.0)	
Feeling lonely (%)	None	1,332 (86.7)	1,371 (89.3)	1,430 (93.1)	1,429 (93.0)	<0.001
	Mild	152 (9.9)	114 (7.4)	81 (5.3)	72 (4.7)	
	Moderate	38 (2.5)	33 (2.1)	13 (0.8)	24 (1.6)	
	Severe	14 (0.9)	18 (1.2)	12 (0.8)	11 (0.7)	
Loss of identity (%)	None	1,536 (100.0)	1,536 (100.0)	1,536 (100.0)	1,536 (100.0)	NA
Feeling hopeful (%)	None	238 (15.5)	556 (36.2)	802 (52.2)	832 (54.2)	<0.001
	Mild	239 (15.6)	199 (13.0)	158 (10.3)	119 (7.7)	
	Moderate	447 (29.1)	307 (20.0)	208 (13.5)	211 (13.7)	
	Severe	612 (39.8)	474 (30.9)	368 (24.0)	374 (24.3)	

Data are expressed as median (interquartile range) or mean (standard deviation) for continuous variables and as count (percent) for categorical variables. Probability was generated based on the *F*-test. NA, not available.

### Symptom changes

**Figure 2:**
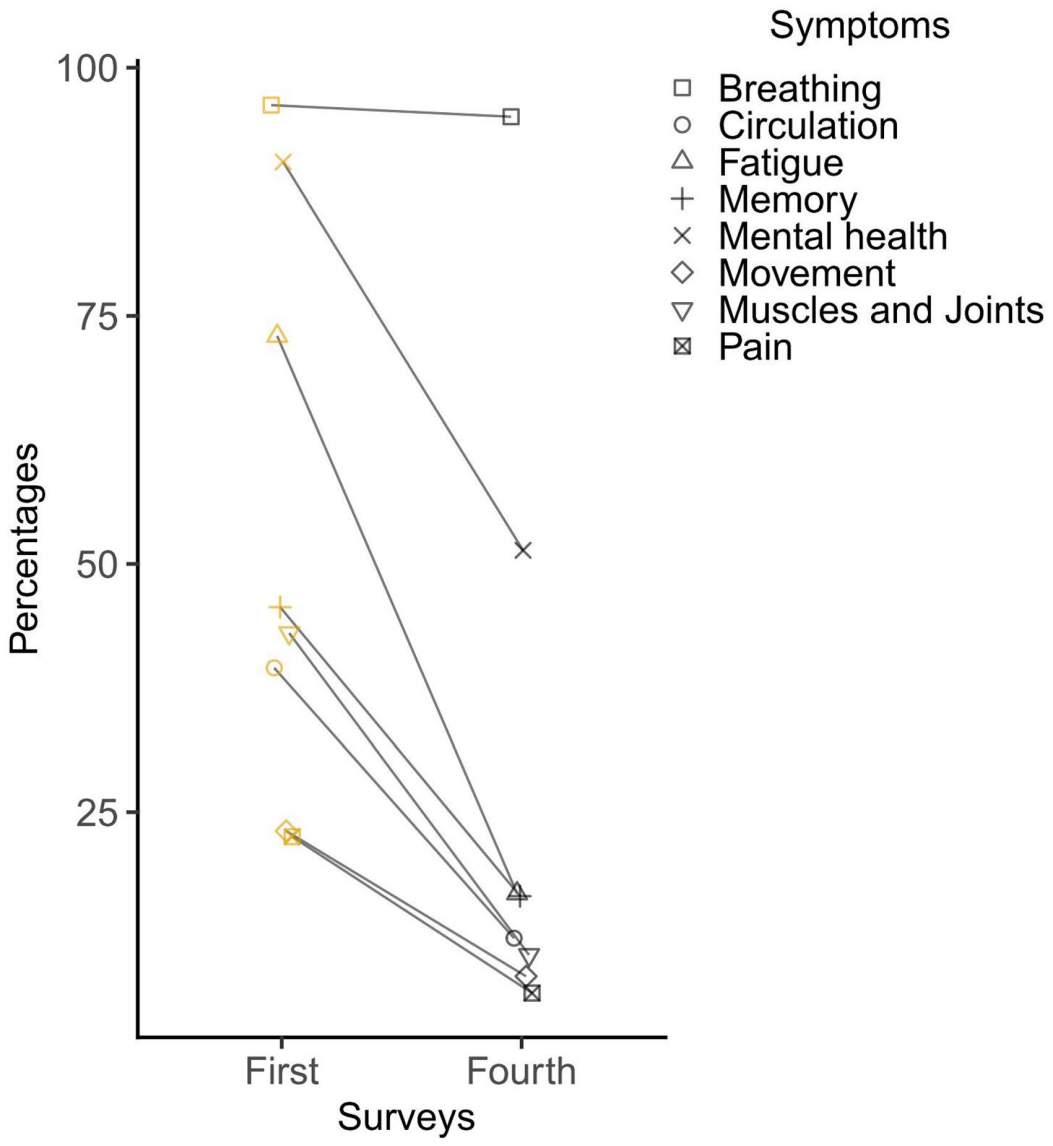
The changes on the presence of post-Omicron symptoms between the first (1st month) and fourth (2nd month) survey.

The changes on the presence of any post-Omicron symptoms between the first and fourth survey are provided in Figure 2. On the first survey following Omicron waves, 96.22% children reported the presence of breathing-related problems, 90.49% mental health-related problems, 72.98% fatigue-related problems, 45.64% memory-related problems, 43.03% muscles and joints-related problems, 39.52% circulation-related problems, 23.11% movement-related problems and 22.53% pain-related problems. On the fourth survey, 95.05 and 51.37% of children still had one or more problems relating to breathing and mental health, respectively, and the percentage of rest six domains was reduced to less than 20%.

### Score comparison of eight domains

**Figure 3:**
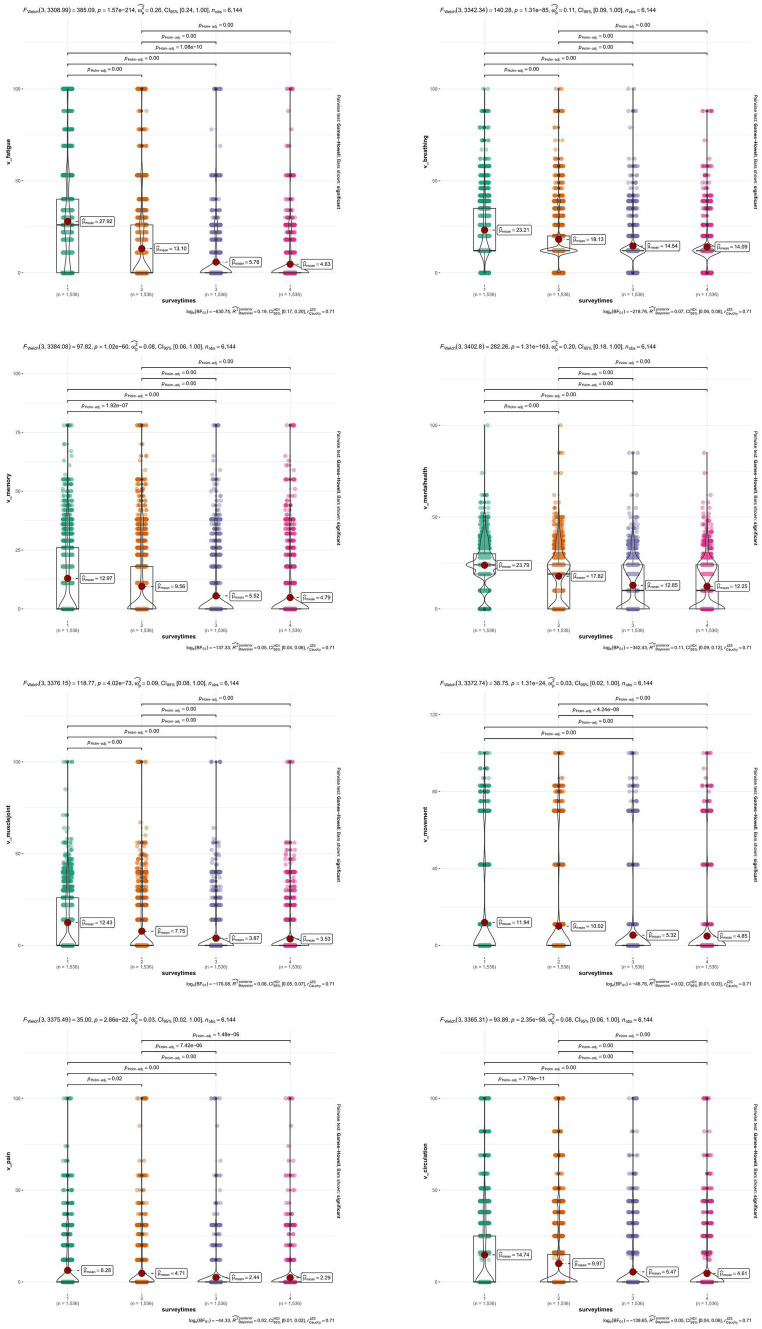
Distribution and comparison of eight domains in the modified SBQ-LC across 4 surveys. SBQ-LC, Symptom Burden Questionnaire for long COVID.

Eight domains were generated based on 51 symptoms, and the raw score of each domain was translated into the Rasch 0–100 linear score according to the User Manual of the SBQ-LC. The distribution of the Rasch linear score for each domain was compared across 4 surveys, as presented in Figure 3. The F-based Welth test showed overall statistical significance of eight domains, and pairwise comparisons across surveys were also significant at a level of 5%.

### Score correlation of eight domains

**Figure 4:**
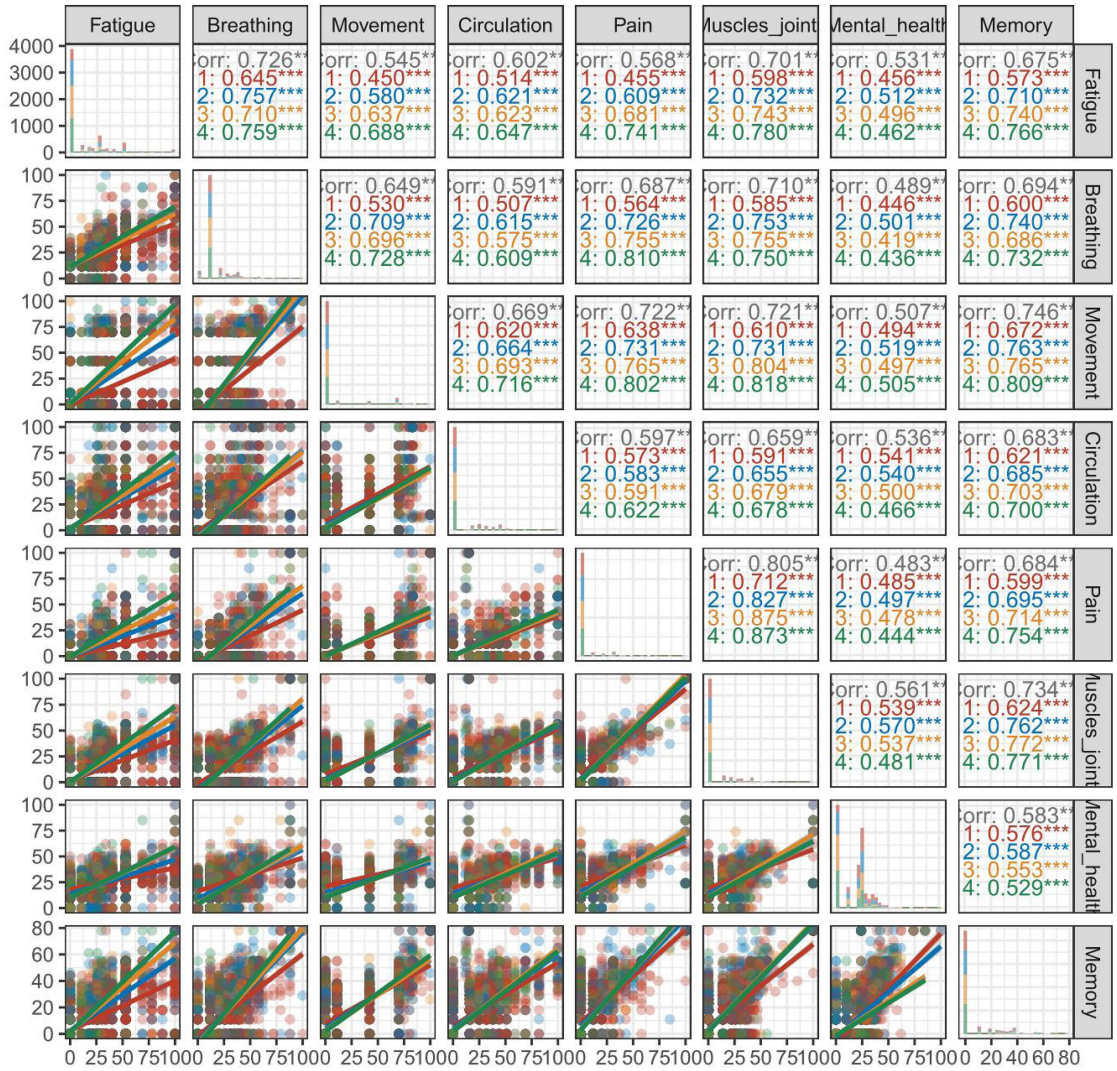
Correlation of eight domains in the modified SBQ-LC stratified by 4 surveys. SBQ-LC, Symptom Burden Questionnaire for long COVID.

The correlation of the Rasch 0–100 linear scores of eight domains across four surveys is illustrated in Figure 4. The overall correlation of eight domains was statistically significant. The correlation of fatigue with movement, pain, muscles and joints, and memory differed obviously across four surveys.

For each survey, the correlation of eight domain scores is illustrated in Supplementary Figure S1. Across the four surveys conducted, a positive correlation was observed for the eight domains. Notably in the fourth survey, the correlation coefficient for mental health with the other domains was found to be relatively weaker than the other comparisons.

### Prediction cubic curves

**Figure 5:**
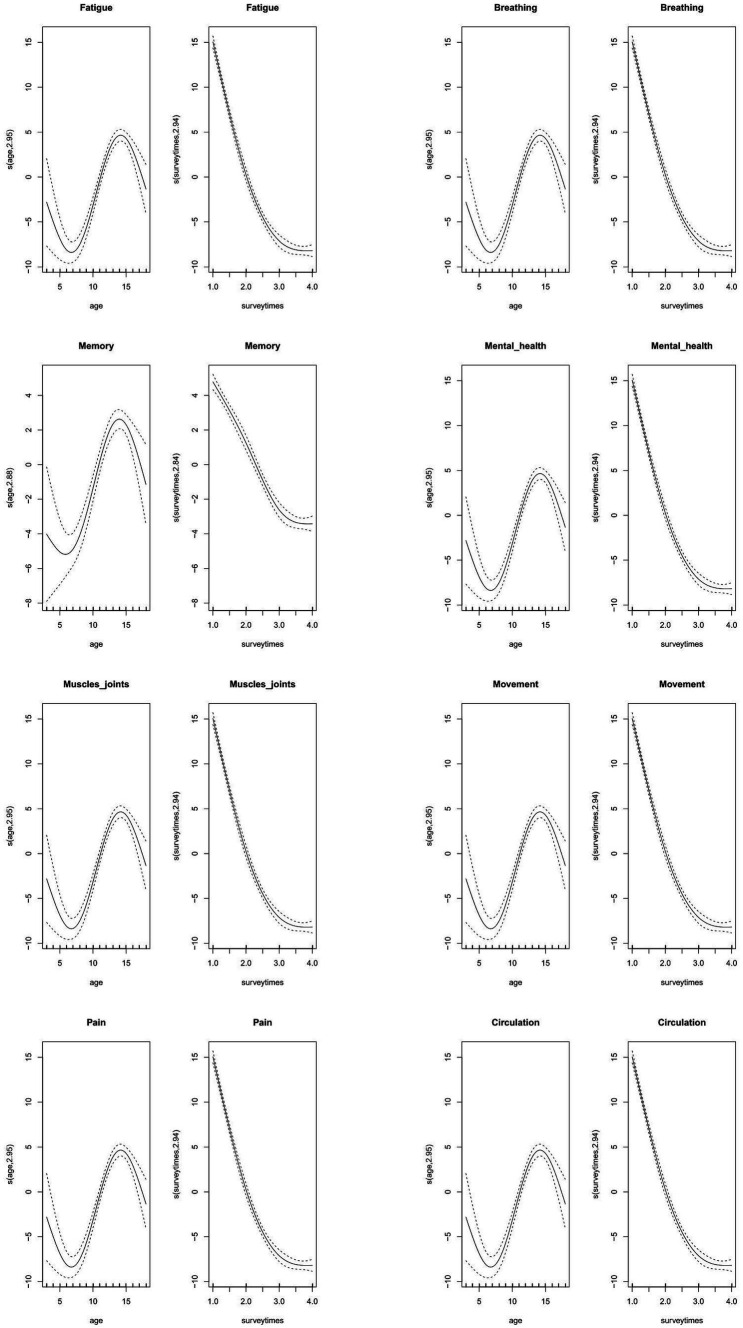
Prediction cubic curves of age and survey for eight domains in the modified SBQ-LC under the generalized additive mixed model. Abbreviations: SBQ-LC, Symptom Burden Questionnaire for long COVID.

The restricted cubic spline smoothed curves for age and survey are provided in Figure 5 for eight domains. Overall, the distribution of age with all domains followed the N-shaped geometry, and that of survey followed the inverse J-shaped geometry. For age, the Rasch linear score hit the lowest level among children aged 6–8 years, and reached the peak among children aged 12–13 years. It is also worth noting that at the 4th survey, 56.95% children had one or more mental health-related problems among children aged 12–13 years, higher than the average level of other age groups at 50.53%. For survey, the scores of all domains sharply declined from the first to the third survey, and remained stable between the third and the fourth survey.

Besides, the combined distribution of age and survey for each domain was also explored under the generalized additive mixed model (Supplementary Figure S2). Eight domains exhibited a saddle-shaped trend with respect to age and survey time, peaking at 12–13 years of age, and all eight domains demonstrated an upward trajectory during the first survey.

### Prediction estimates

Considering the autocorrelational structure of eight domains under study, generalized additive mixed model was adopted and it is an extension of the generalized linear mixed model. Two factors were smoothed in this study, that is, age and survey, and sex was treated as nonsmoothed factor. As shown in Table 2, the association of sex was statistically significant with pain only. Age was associated with all domains at a significance level of 5%, and besides muscles and joints (*p* = 0.058), survey with the other seven domains was significant.

**Table 2:** Association of sex, age and survey with eight domains in the modified SBQ-LC under generalized additive mixed model.

Eight domains in the modified SBQ-LC	Factors	Coef.	SE	*t*	*p*
Fatigue	Sex	0.59	0.66	0.89	0.375
	Age	−7.66	1.76	−4.35	<0.001
	Survey	−9.10	0.72	−12.70	<0.001
Breathing	Sex	0.43	0.48	0.89	0.371
	Age	−4.00	1.23	−3.25	0.001
	Survey	−2.85	0.45	−6.35	<0.001
Movement	Sex	0.43	0.48	0.89	0.371
	Age	−4.00	1.23	−3.25	0.001
	Survey	−2.85	0.45	−6.35	<0.001
Circulation	Sex	0.43	0.48	0.89	0.371
	Age	−4.00	1.23	−3.25	0.001
	Survey	−2.85	0.45	−6.35	<0.001
Pain	Sex	2.43	0.65	3.72	<0.001
	Age	−7.25	1.70	−4.25	<0.001
	Survey	−2.79	0.58	−4.80	<0.001
Muscles and joints	Sex	−0.32	0.49	−0.65	0.514
	Age	−3.67	1.20	−3.06	0.002
	Survey	−0.69	0.36	−1.90	0.058
Mental health	Sex	−0.65	0.54	−1.19	0.233
	Age	−3.92	1.37	−2.86	0.004
	Survey	−2.42	0.46	−5.25	<0.001
Memory	Sex	0.55	0.48	1.16	0.248
	Age	−4.93	1.25	−3.95	<0.001
	Survey	−3.12	0.47	−6.71	<0.001

SE, standard error.

### Survey-dependent score changes of eight domains

The survey-dependent changes of the Rasch scores of eight domains are provided in Supplementary Figure S3. The difference between the first and second survey across eight domains was predominantly increased, the difference between the second and third survey has comparable ups and downs, and the difference between the third and fourth survey was predominantly decreased.

### Symptom combinations

The symptom combinations of each domain across 4 surveys are displayed in the form of Venn plots in Supplementary Figures S4–S7, respectively. In the first survey, 51.8% of children reported the simultaneous presence of four symptoms in fatigue domain; 44.4 and 44.5% of children reported one symptom in breathing and mental health domains; 76.9 60.5, 77.5, 56.9, and 54.4% of children reported no presence of any symptom in movement, circulation, pain, muscles and joints and memory domains. In the second survey, 65.6 and 43.1% of children reported one symptom in breathing and mental health domains; 62.6, 82.7, 74.3, 85.5, 75.6, and 68.9% of children reported no presence of any symptom in fatigue, movement, circulation, pain, muscles and joints and memory domains. In the third survey, 78.8% of children reported one symptom in breathing domain; 79.8, 90.6, 84.5, 92.7, 87.9, 46.2, and 81.1% of children reported no presence of any symptom in fatigue, movement, circulation, pain, muscles and joints, mental health and memory domains. In the fourth survey, 81.6% of children reported one symptom in breathing domain; 83.2, 91.5, 87.7, 93.2, 89.4, 48.6, and 83.5% of children reported no presence of any symptom in fatigue, movement, circulation, pain, muscles and joints, mental health and memory domains.

## Discussion

The aim of this prospective study was to assess the dynamic changes of persisting symptoms among children aged 6–18 years during 1–2 months after the Omicron infection based on the modification of SBQ-LC from eight domains in Beijing, China. The key findings of this study are that the impact of Omicron infection on the health of children is multifaceted, and of eight domains evaluated, breathing- and mental health-related problems persisted during 1-to-2-month Omicron infection period. Moreover, the impact of Omicron infection on the health of children peaked among children aged 12–13 years, who are experiencing the transition from primary school to junior high school in practice. To hasten post-COVID-19 recovery, special attention should be given to vulnerable age groups who had the highest risk of detrimental multisystem symptoms. As far as we know, this is thus far the first prospective survey on post-Omicron symptoms among Chinese children in the literature.

It is universally accepted that children, as a special group, are very sensitive and vulnerable, and warrant special care, especially when facing regional or global serious pandemics, such as COVID-19. Global statistics show that of all confirmed cases, pediatric COVID-19 is estimated to account for 2–7%. Currently, there is wide recognition that in the long-term, COVID-19 has left a large proportion of adults and children suffering unexplained persistent residual or new-onset symptoms that may affect routine functioning and fluctuate or relapse over time (12–15). These symptoms, collectively termed as long COVID, are prevalent and present among 25% of children according to a meta-analytical report (8). It is clearly important to achieve a better understanding of the symptoms that prevail during the long COVID period, so that preventive or interventive measures may be put in place to improve quality of life and reduce the burden of disease and disability. At present, evidence on post-Omicron symptoms mainly draws from mono cross-sectional surveys and outpatient or inpatient patients (10, 16–20), and the dynamic picture of these symptoms, especially following Omicron waves, is not fully understood in children from China. To shed some light, we designed this prospective study by monitoring the 1-to-2-month changes of post-Omicron symptoms of 1,536 children aged 6–18 years from Tongzhou district, Beijing at serial even intervals.

In this study, it is worth noting that the impact of Omicron infection on the health of children involves multiple systems, in particular breathing and mental health with over 90% children suffering one or more relevant symptoms one-month post-Omicron, even after 3 months, one or more these symptoms persisted among over half children. Persisting breathing-related problems are very easy to trigger the development of lower respiratory tract infection, an important cause of children morbidity and mortality around the globe (21). Moreover, systematic evidence suggests that children with confirmed lower respiratory tract infection during the first 3 years of life often have abnormal pulmonary function testing and are susceptible to obstructive airways diseases (22, 23). As such, our public health decision-makers and healthcare professionals working in primary care settings should attach much emphasis to the respiratory health of children following Omicron waves. Our findings underscore the need for enhanced comprehensive monitoring on the patterns and progressions of breathing-related symptoms in practice, as well as the need for early identification and targeted intervention of high-risk children for lower respiratory tract infection and pneumonia to reduce the escalating burden of COVID-19 pandemic globally. Besides, there is evidence that COVID-19 infection can accelerate the adverse psychological and behavioral outcomes, and a wide range of brain changes were reported in patients with pediatric mental illness (24). Other studies have shown that mental health continued to deteriorate through the COVID-19 pandemic even after easing restrictions (25). According to our recent report, longtime home study was associated with a high rate of psychological and behavioral problems in children after the COVID-19 pandemic, and this rate reached 13.51% in children aged 6–11 years and 18.08% in children aged 12–16 years (26). Further, we found that reasons behind these psychological and behavioral problems might be attributable to parent–child conflict, sleep problems, prolonged online study time and video-game time and increased sedentary time (27). At the time of our serial surveys, children infected with COVID-19 were required to study at home again, with their psychological behaviors facing great challenges. Hence, the implication of this study is a call for action to pay special attention to mental health in children after the Omicron infection.

Another important finding of this study is that the detrimental impact of Omicron infection in childhood health was largest among children 12–13 years of age especially in terms of breathing and mental health, who are undergoing the transition from primary school to junior high school during the period from 2019 to 2022 in Beijing. It is generally accepted that children of this age group are experiencing periodical changes in physiological, mental and behavioral aspects throughout the human lifespan. These changes are potentially worsened when facing the repeated COVID-19 outbreaks. We all agree that compulsory education builds a foundation upon which every aspect of children’s development relies, yet studying online at home disrupts normal education, prevents children from getting their education off the best possible start, and reduces face-to-face communication as well. As stated by Sun and colleagues, China needs a scientific long COVID recovery-support platform, which can help the public understand the symptoms and effects of long COVID, and provide guidance to patients on how to cope with symptoms during their recovery (28). In the post-pandemic era, childcare is hence essential in providing children with integrated services and protection, as well as developing social, emotional and cognitive skills, especially for children aged 12–13 years.

The findings of this prospective study provide schools and healthcare professionals with valuable information, as reflected in the following aspects. First, 1–2 weeks are required for children to adjust and adopt the early stage of reopening schools. Second, the intensity of physical and school assignment objectives should be increased gradually according to the status of children. Third, mental health-related problems should be closely monitored and channeled out in time via the integrated efforts of both parents and teachers.

Some limitations should be acknowledged for this study. First, our questionnaire was designed by referring to the SBQ-LC version 1.0, which is suitable for adults only and constructed from 16 domains. Contrastingly, we only adopted 8 domains suitable for children, and whether doing so can be applicable deserves special concerns, as no official questionnaires are currently available for children under long COVID. Second, only data on four serial surveys following Omicron waves were available for the present study, and considering the persisting health-related problems, further longitudinal surveys, which are ongoing, necessitate. Third, only data on eight groups of clinical symptoms were collected, and as incorporating dynamic lung imaging and viral nucleic acid CT values would have been more meaningful, this substantially weakened the results. Fourth, study children were enrolled from the Tongzhou district, Beijing, and whether our regional observations can be extrapolated to other regions warrants further validation. What’s more, all children are of Chinese origin, and generalizability to other racial or ethnical groups should be done with caution.

Despite above potential limitations, our findings indicate the multifaceted impact of Omicron infection on childhood health, especially among children aged 12–13 years. Moreover, of eight domains evaluated, breathing and mental health related problems persisted during 1–2-month Omicron infection period and the rest six domains were significantly improved. Importantly, the implication of this study is a call for action to build a long COVID recovery-support platform for children around the world, especially in the domain of respiratory and mental health and among children currently attending junior high schools.

## Data Availability

The raw data supporting the conclusions of this article will be made available by the authors, without undue reservation.

## Footnotes

^1^ https://www.wjx.cn/
