# Supplementary material for: Impact of Omicron infection on childhood health: the Beijing long-COVID study
Source: Front Public Health. 2025 Feb 19;13:1377745. doi: 10.3389/fpubh.2025.1377745 (PMC11879840; doi:10.3389/fpubh.2025.1377745)

**Supplementary Figure 1.** Correlation of eight domains in the modified SBQ-LC across four surveys.

**1^st^ 2^nd^**


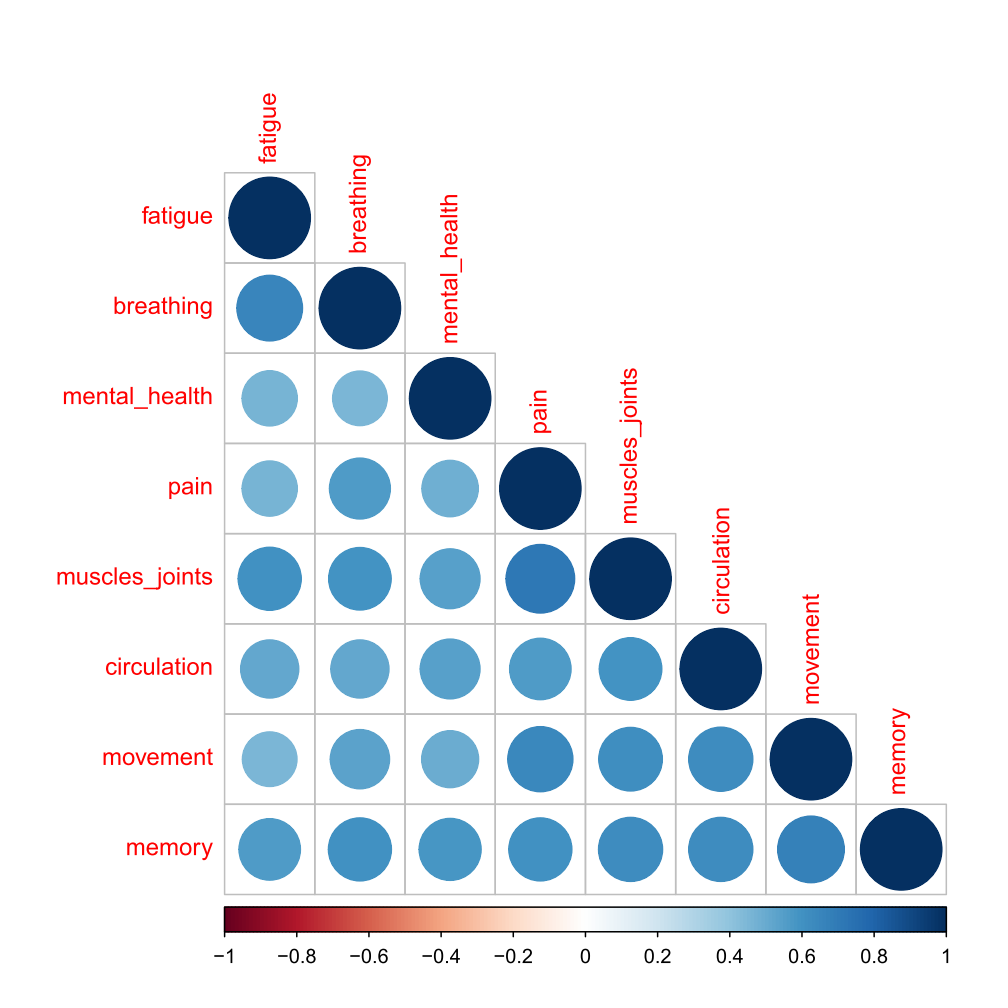

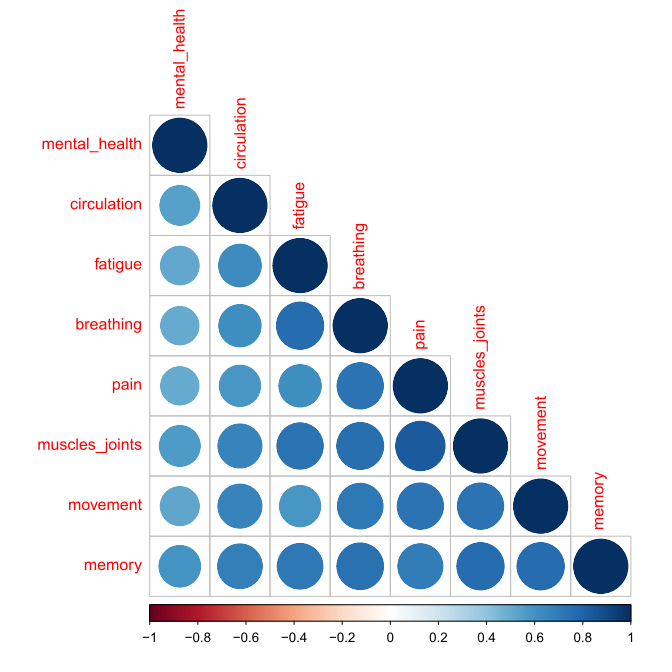


**3^rd^ 4^th^**


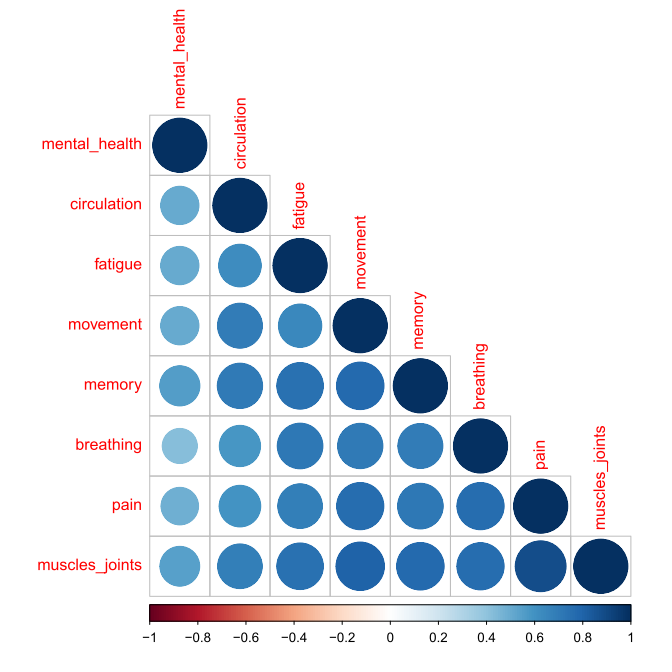

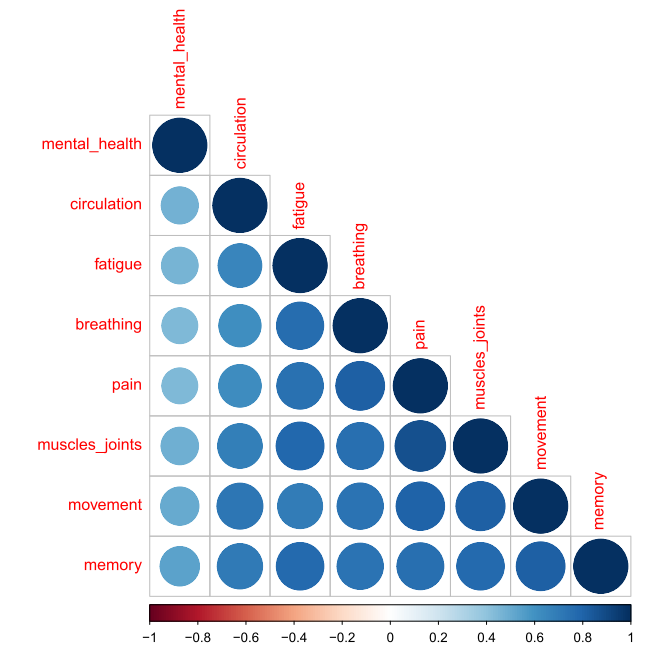


**Supplementary Figure 2.** Combined distribution of age and survey for eight domains in the modified SBQ-LC under the generalized additive mixed model.


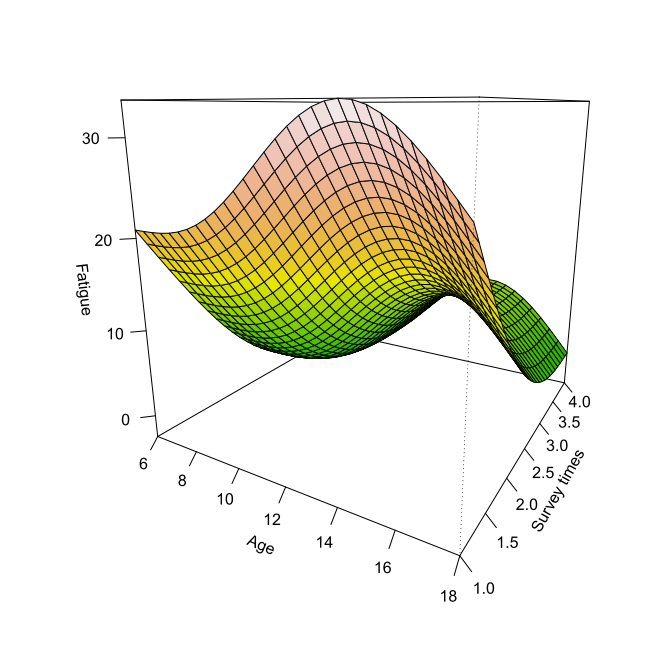

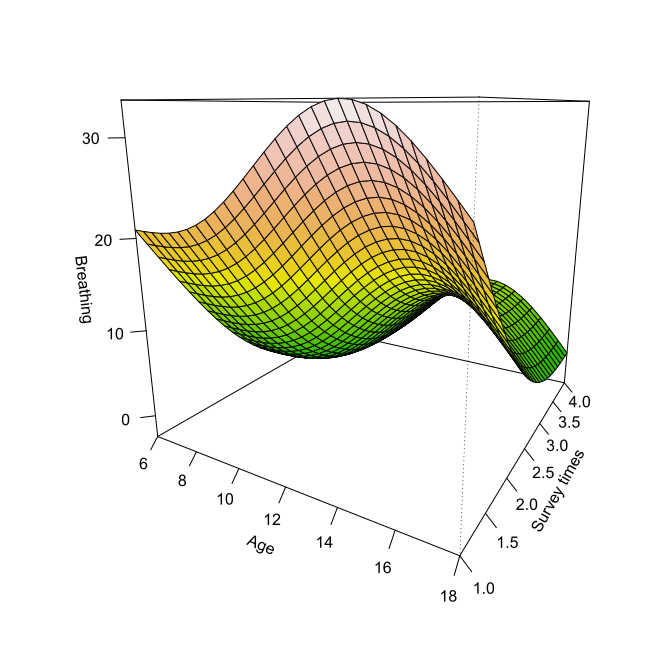


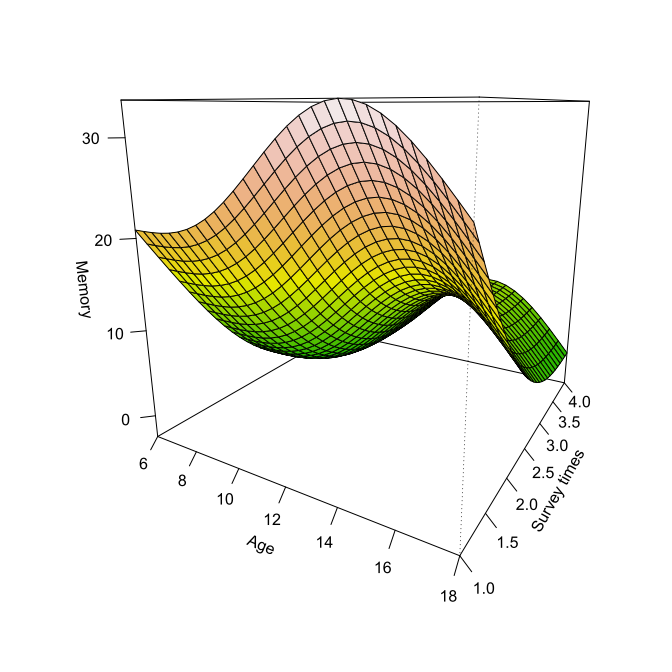

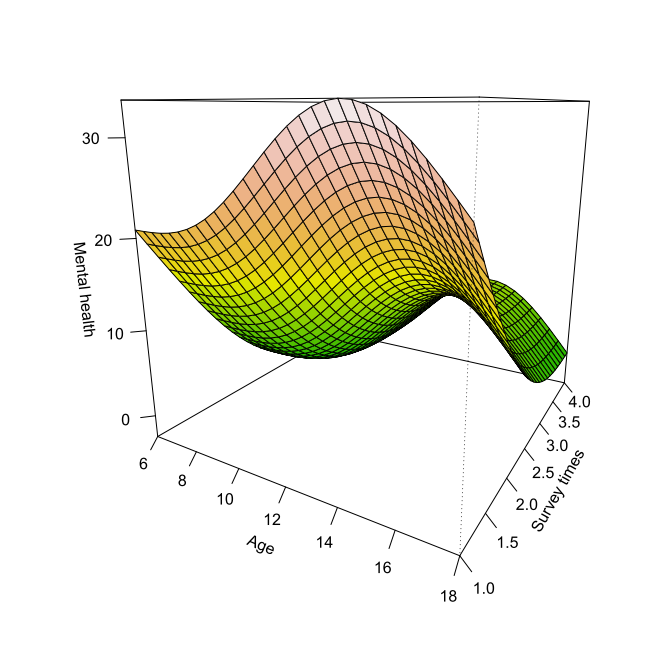


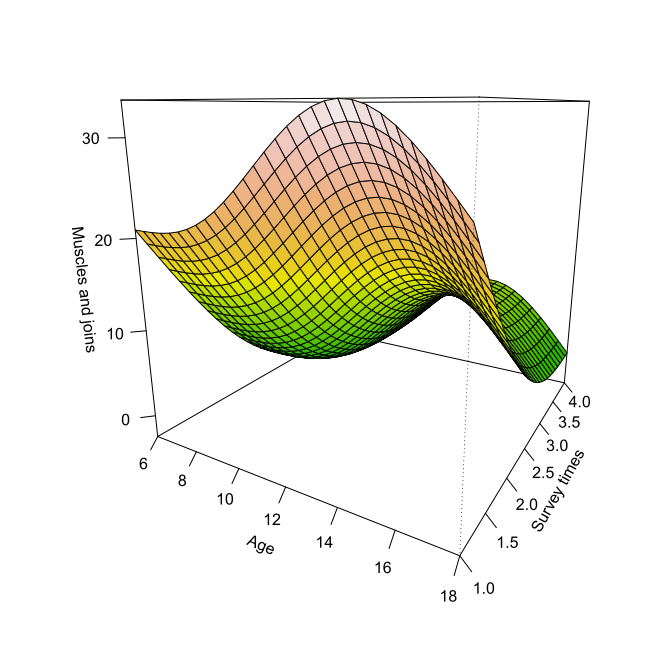

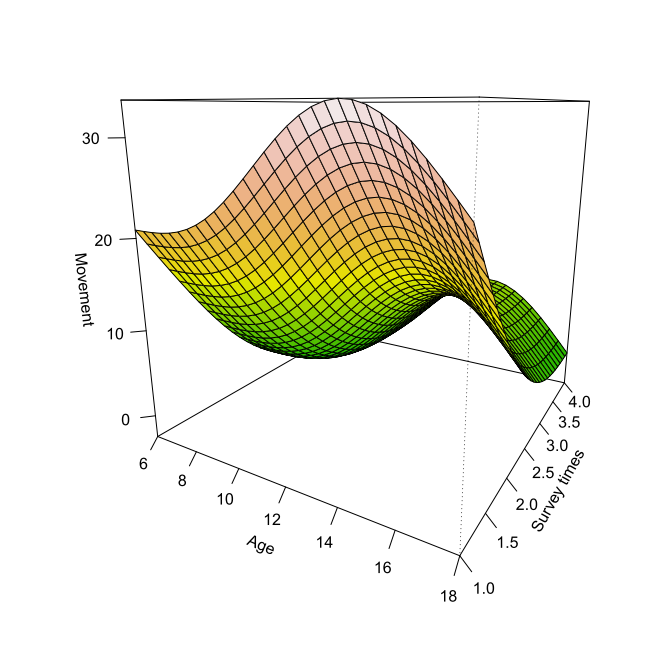


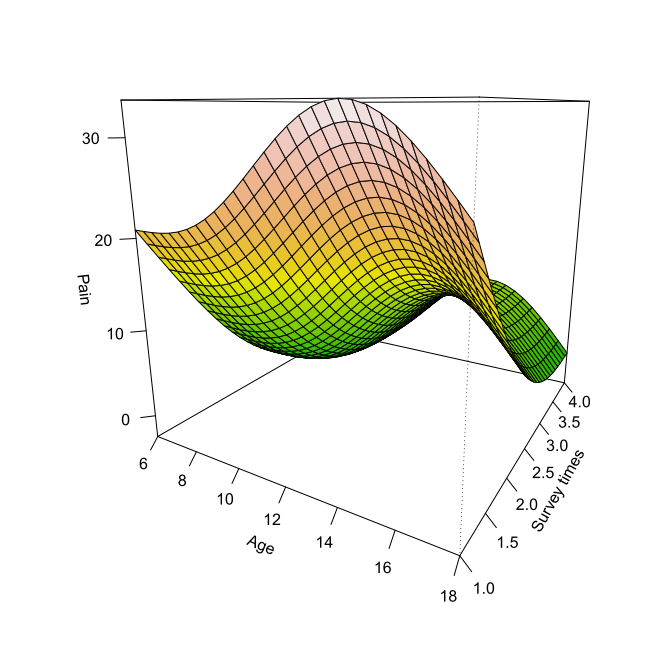

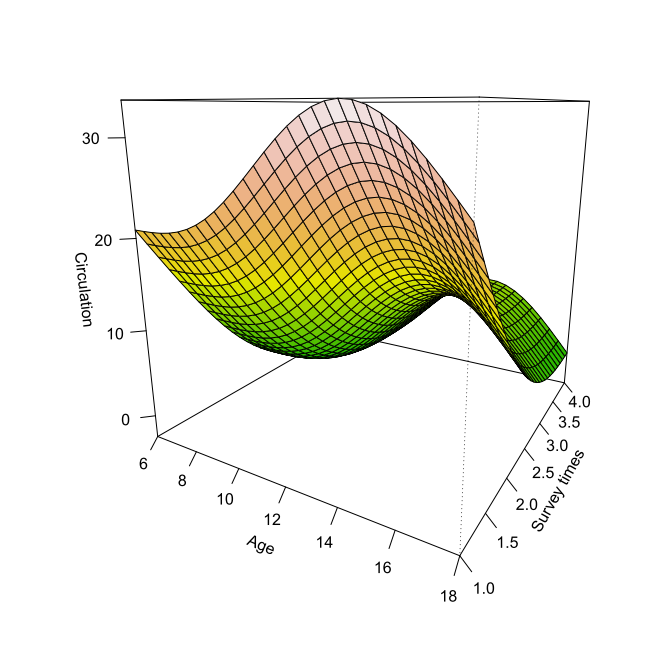


**Supplementary Figure 3.** Changes in eight domains in the modified SBQ-LC across 4 surveys. The change ended with number 1 denotes the difference between 1^st^ survey and 2^nd^ survey, with 2 the difference between 2^nd^ survey and 3^rd^ survey, and with 3 the difference between 3^rd^ survey and 4^th^ survey.


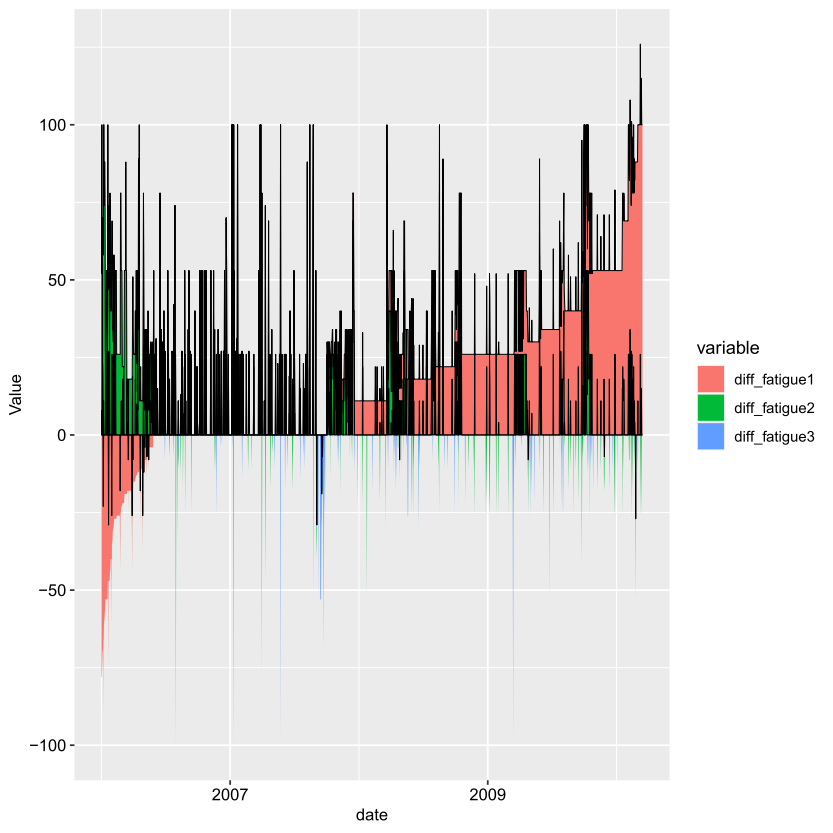

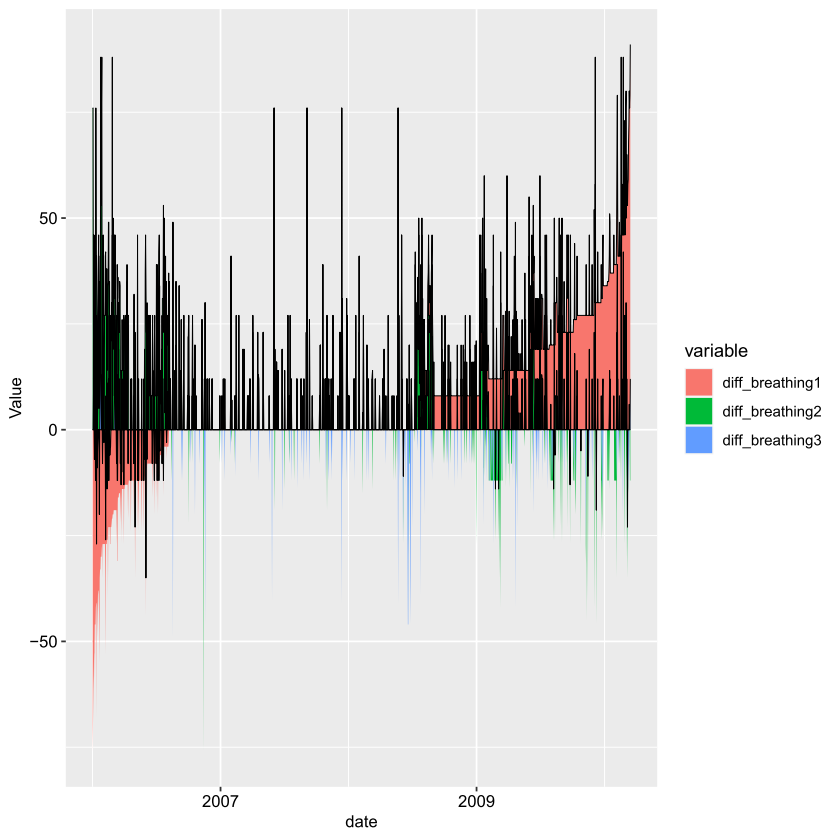


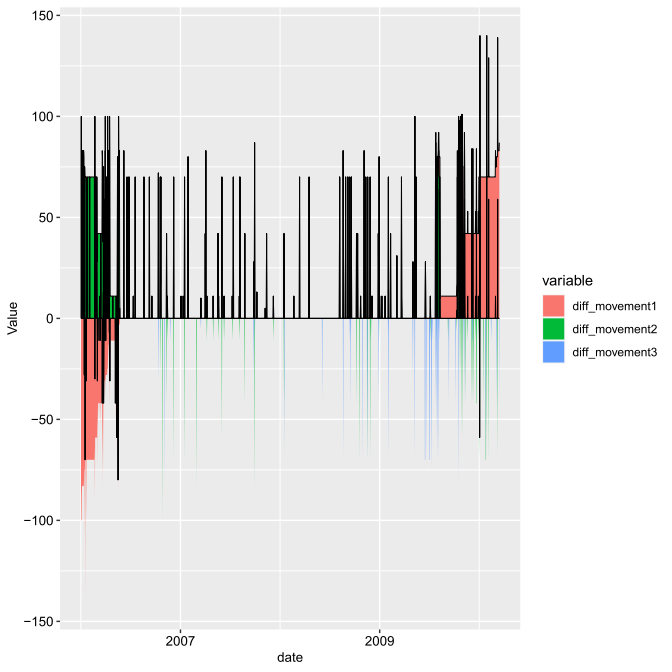

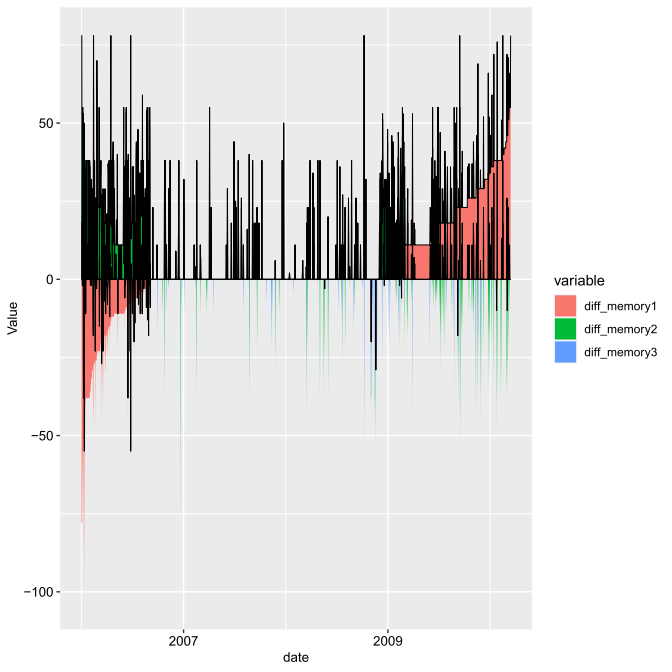

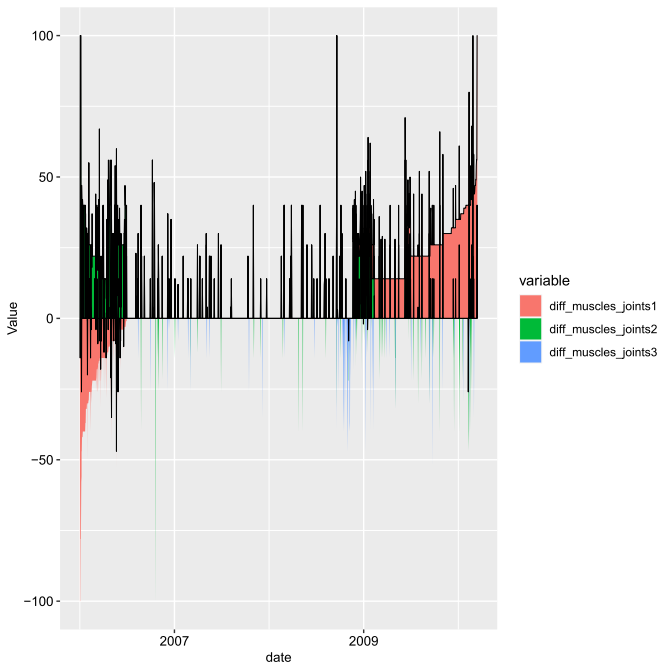

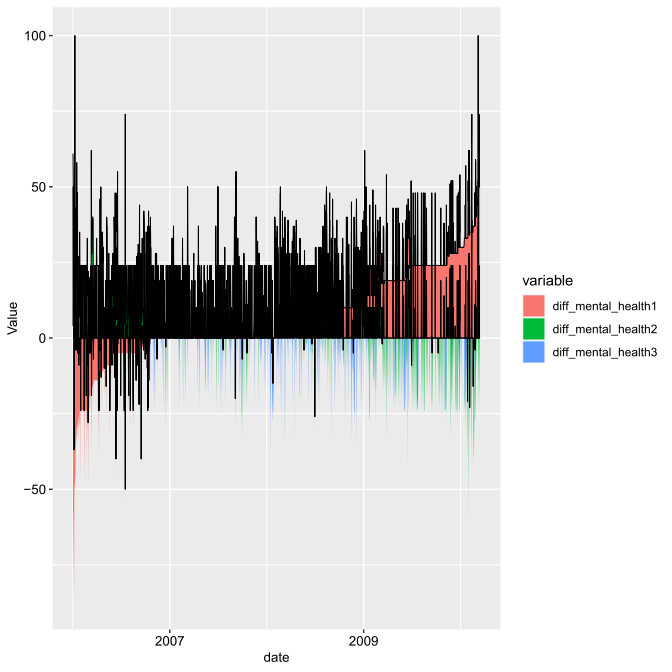

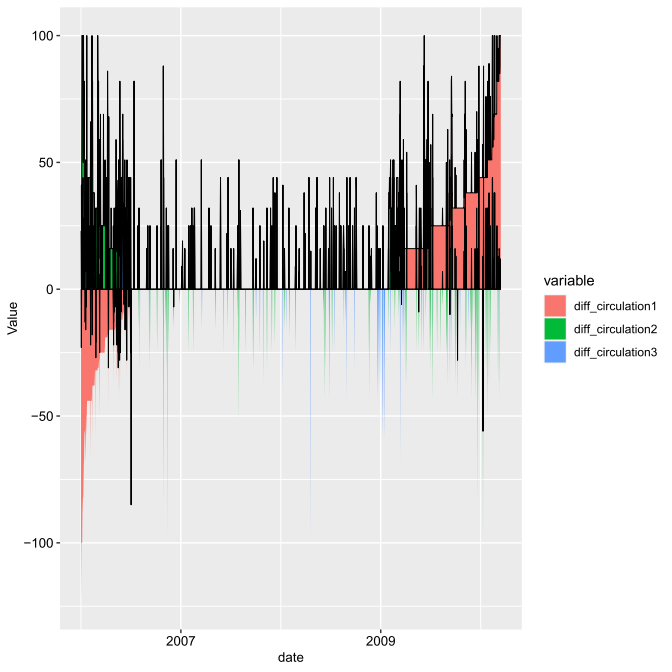

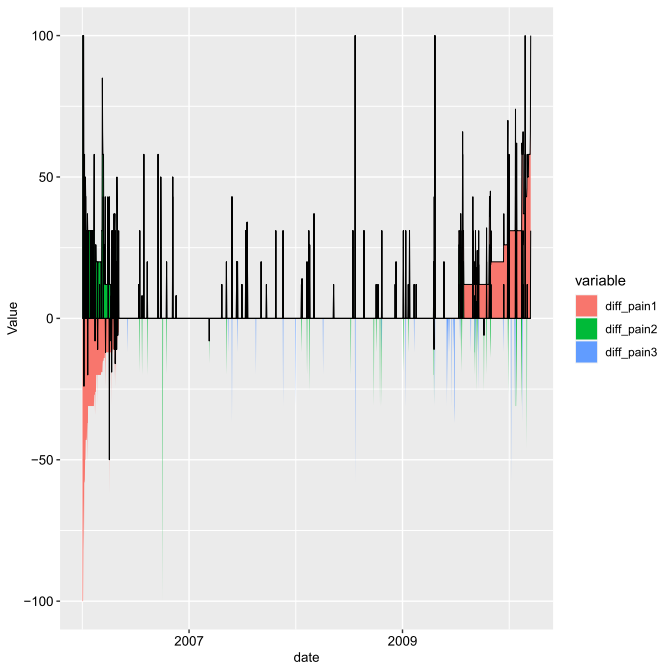


**Supplementary Figure 4.** Distribution of symptom combinations within each domain in the first survey.


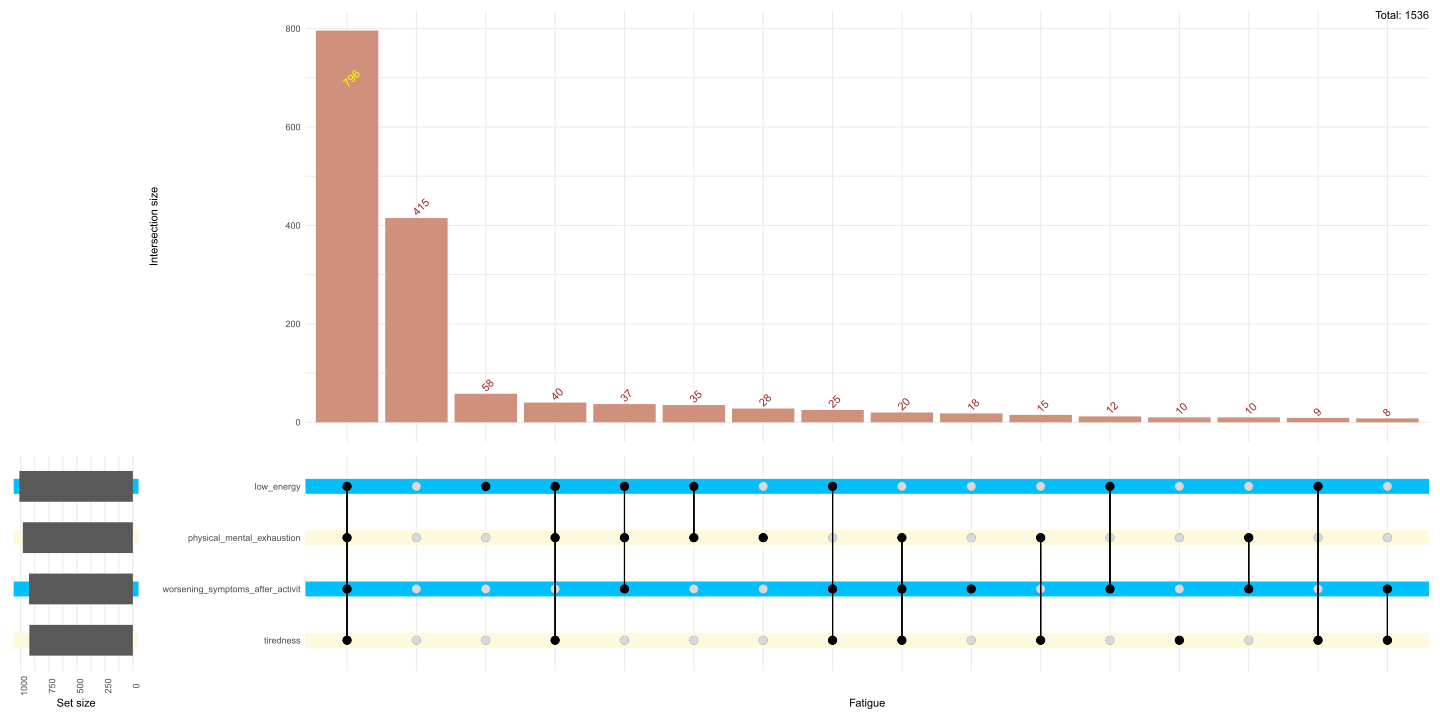


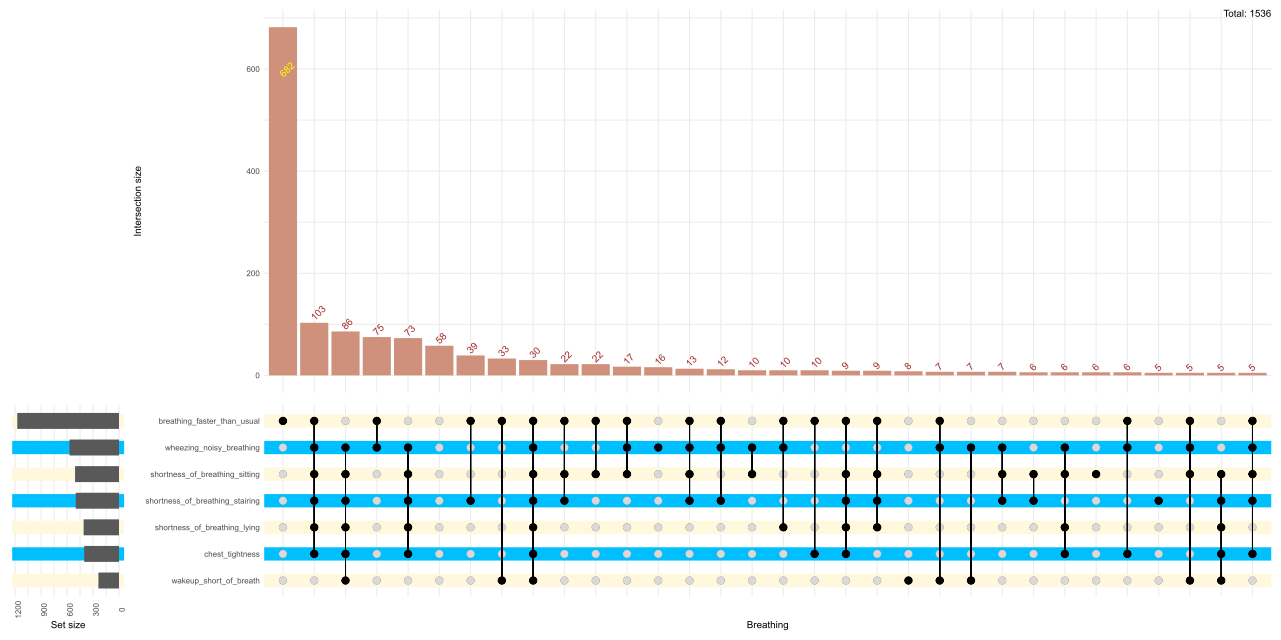

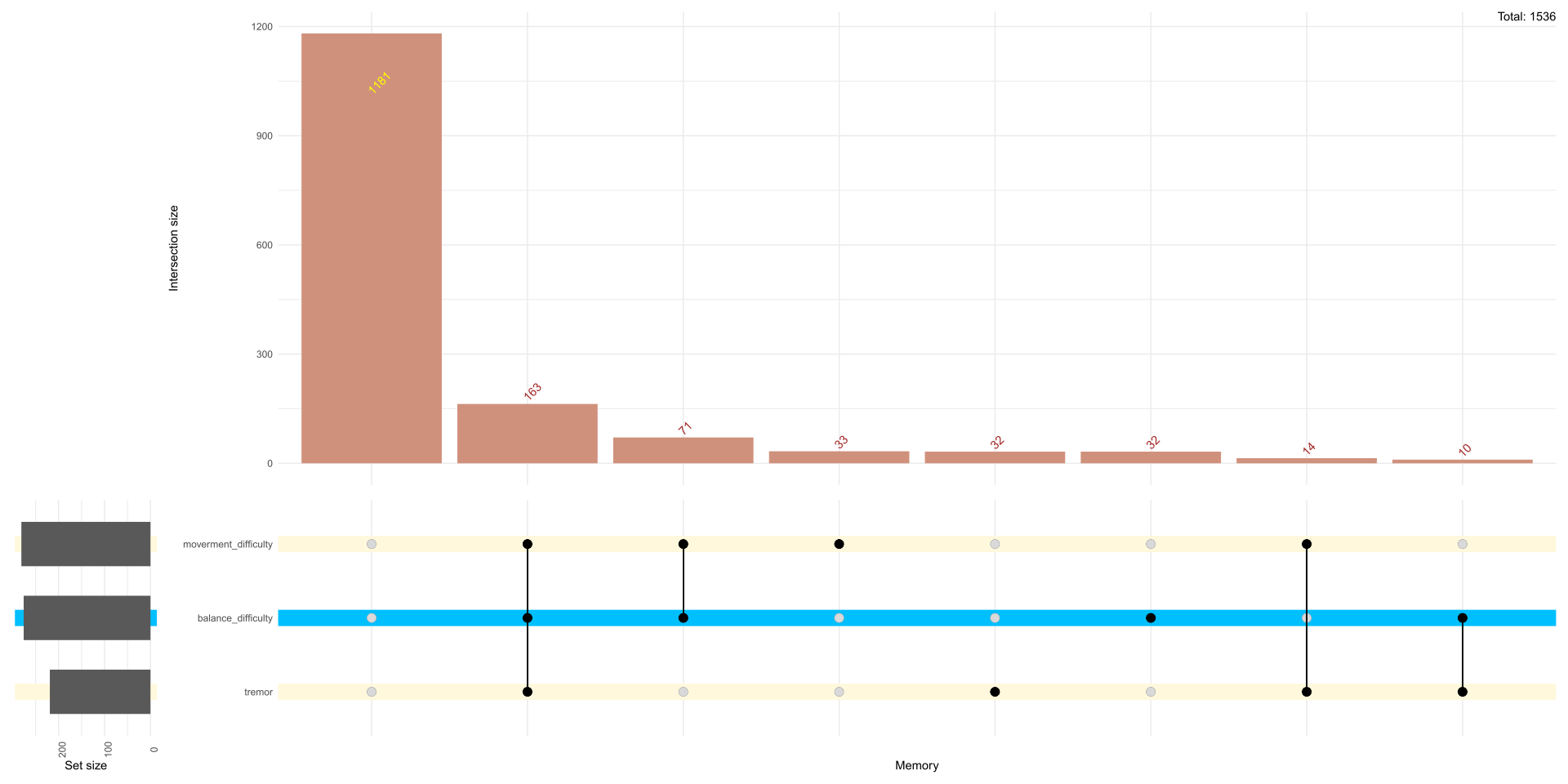

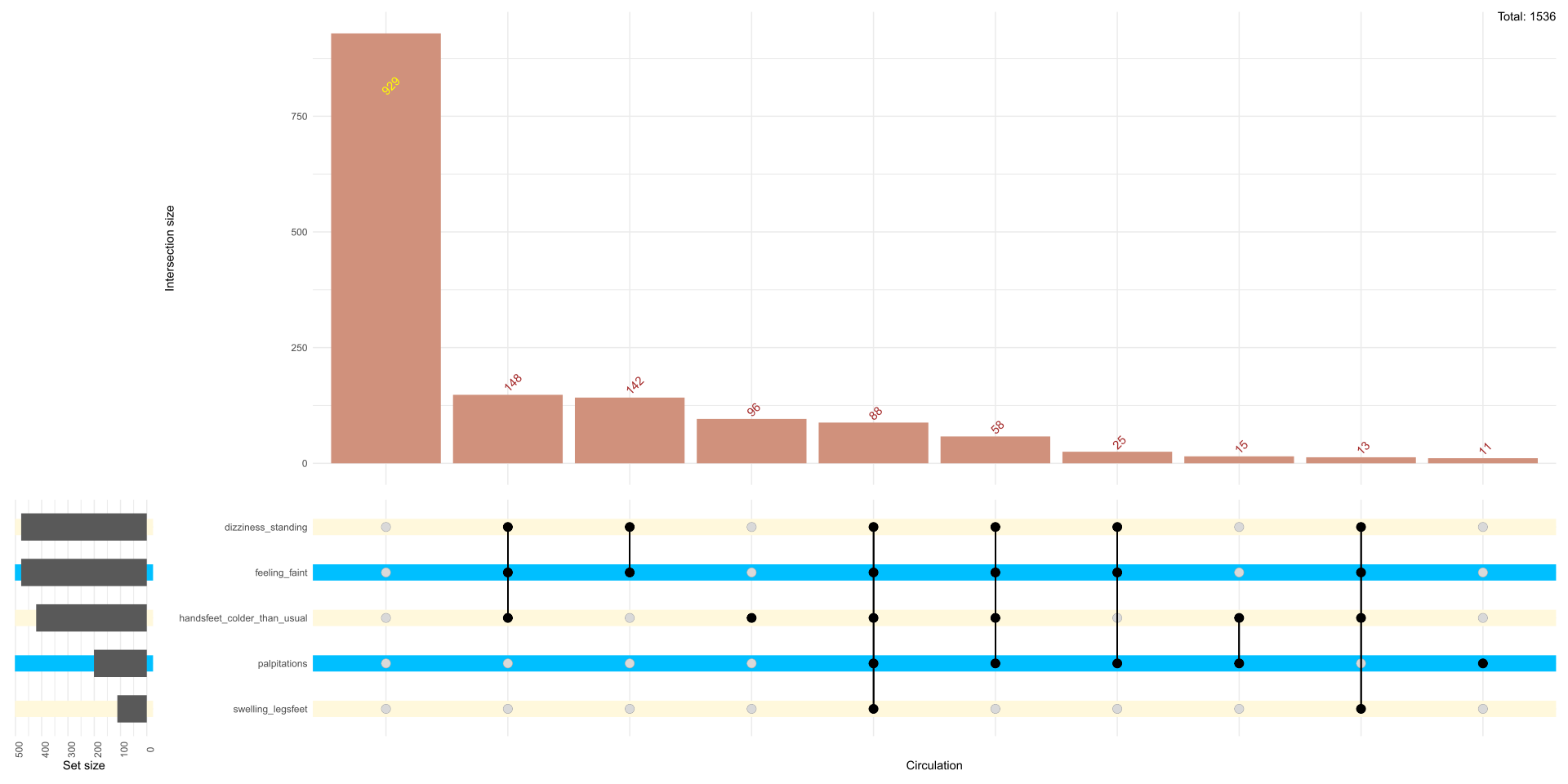

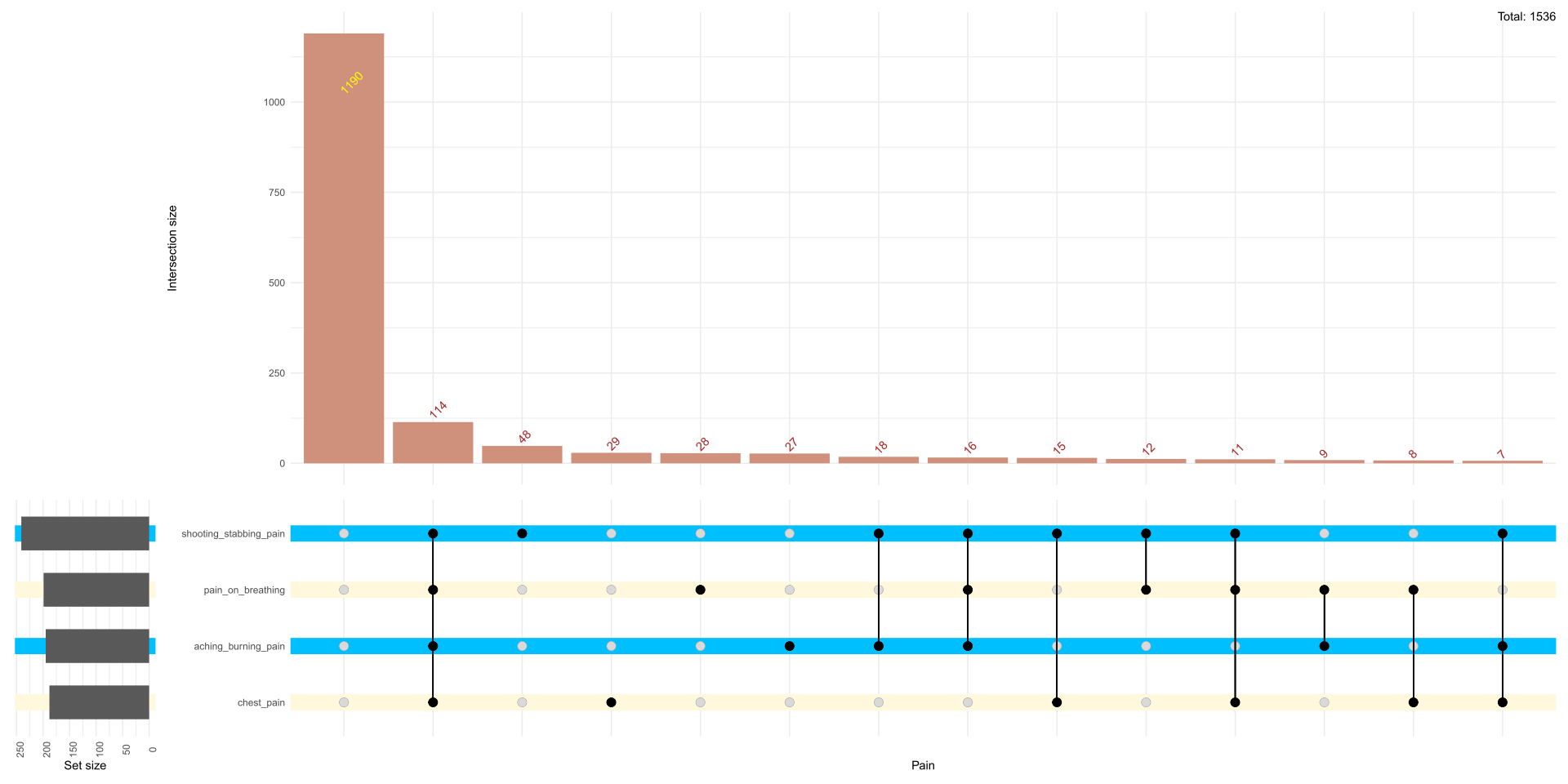

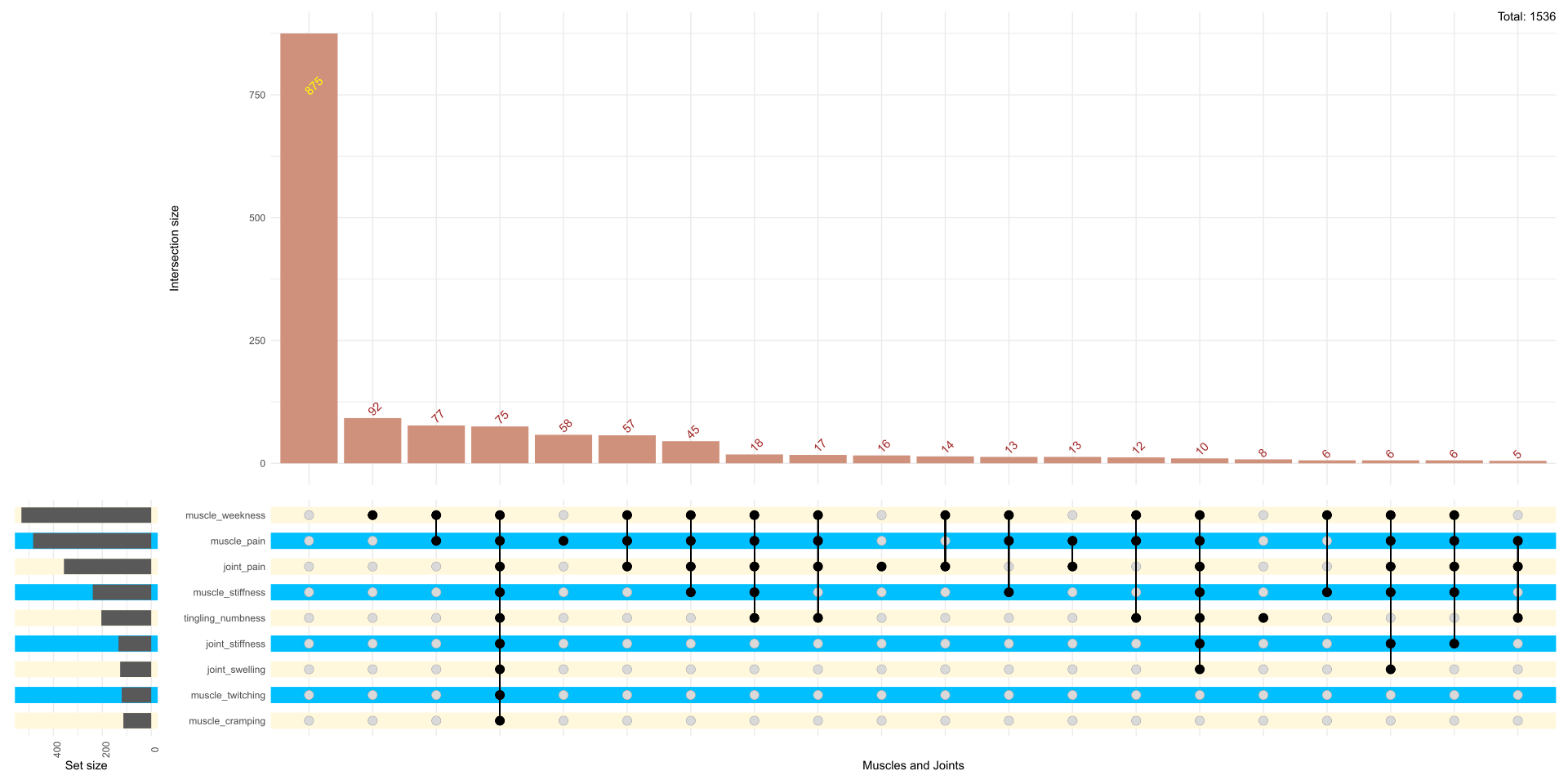

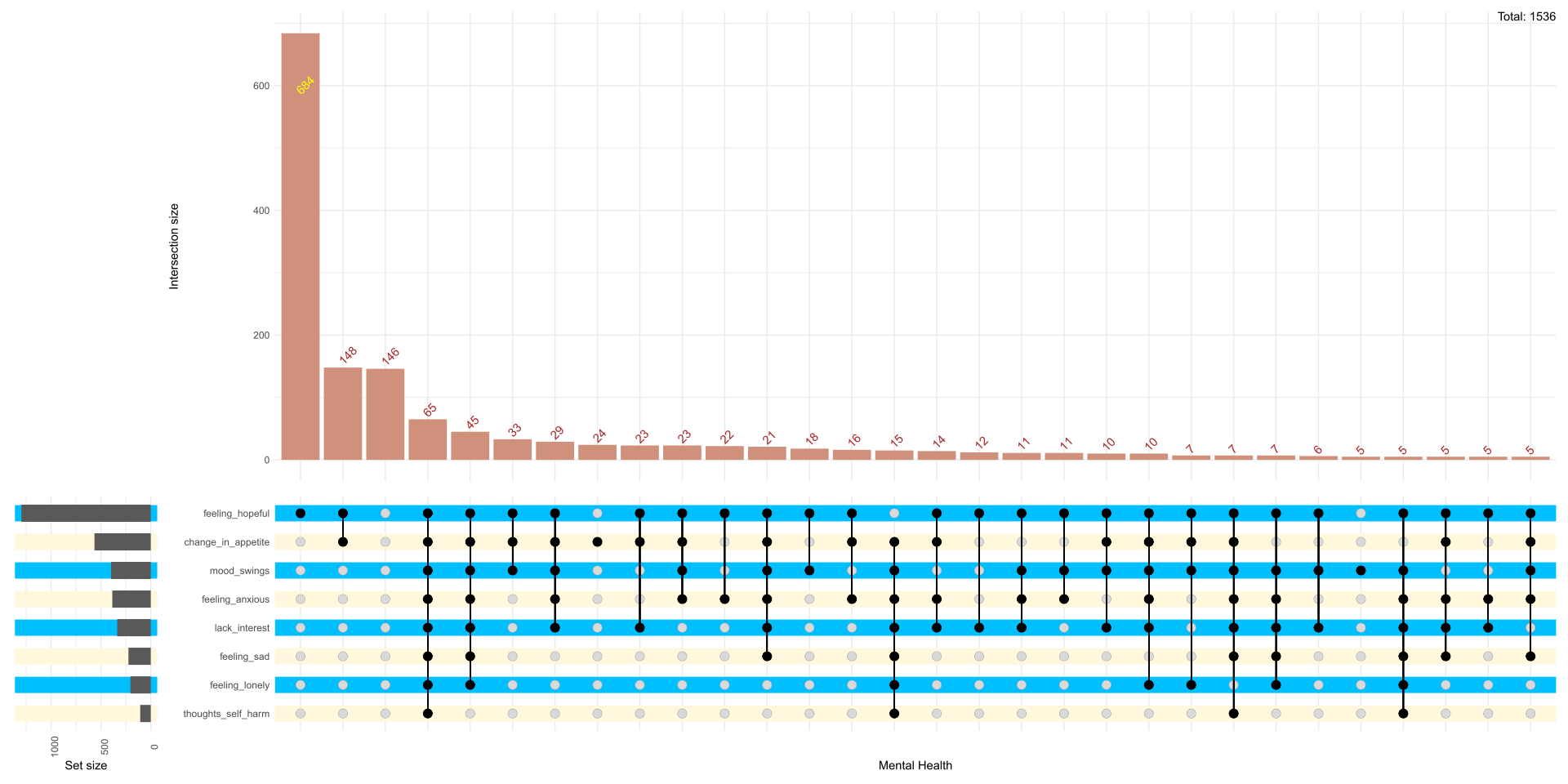

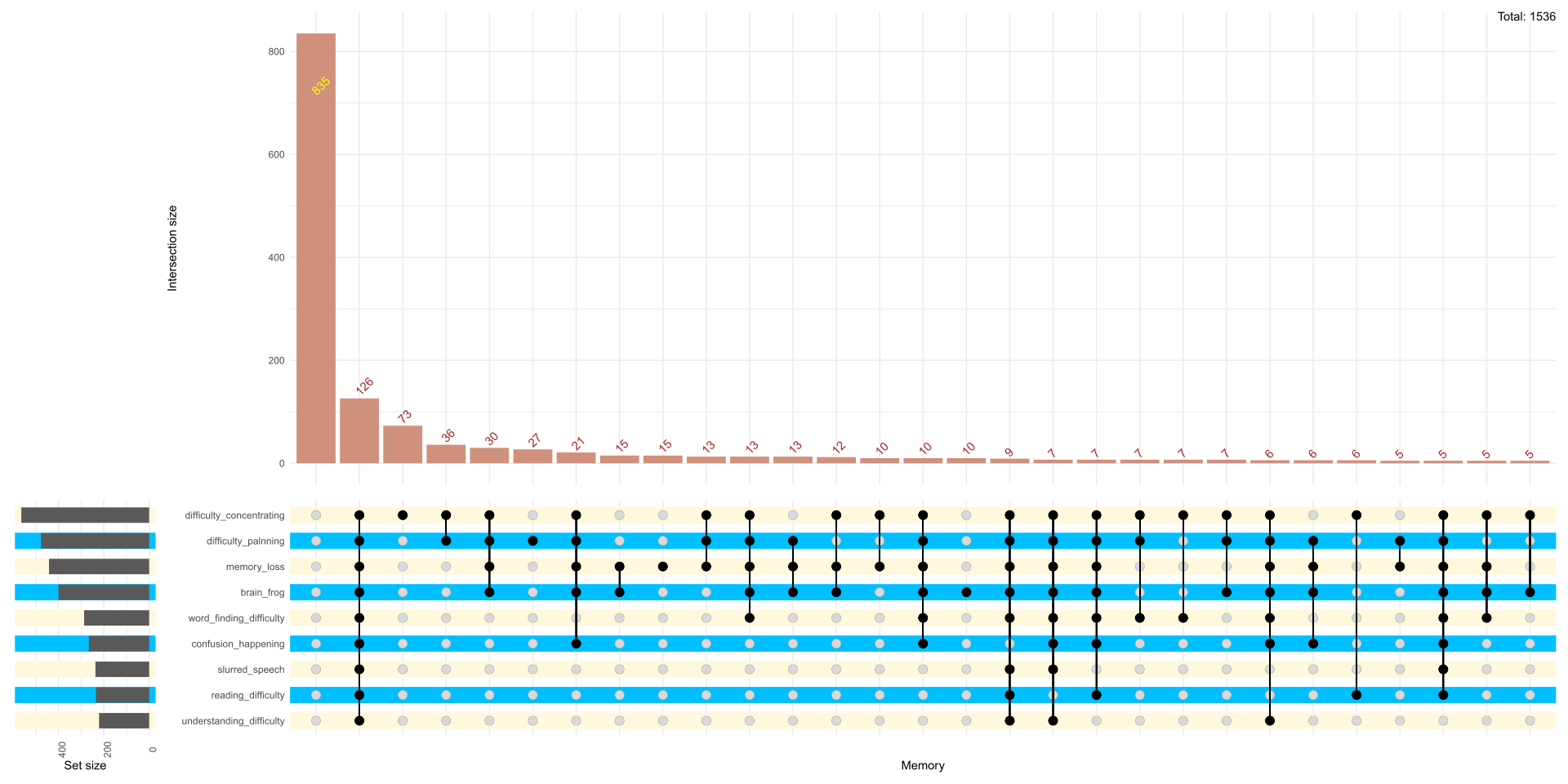


**Supplementary Figure 5.** Distribution of symptom combinations within each domain in the second survey.


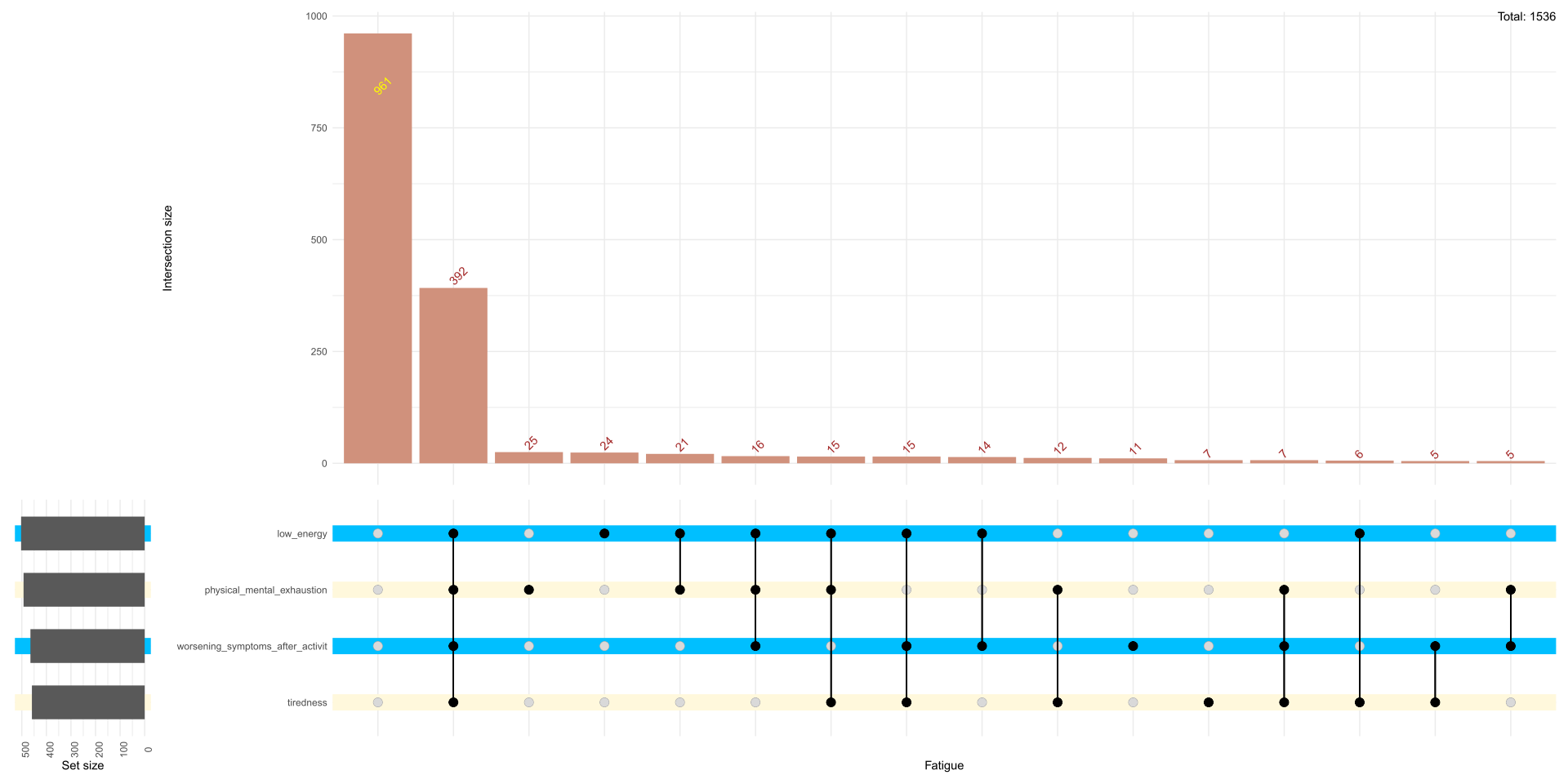


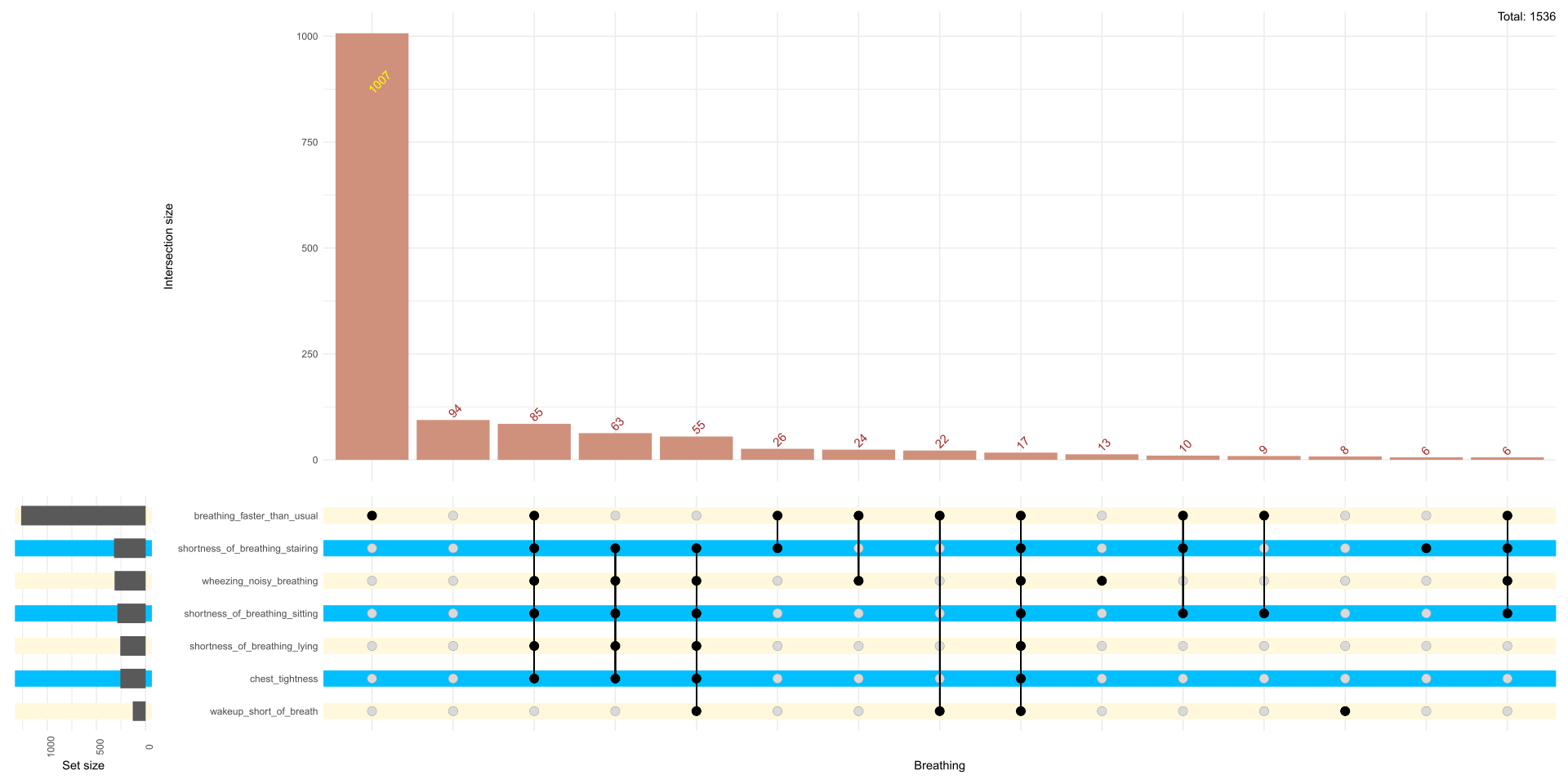

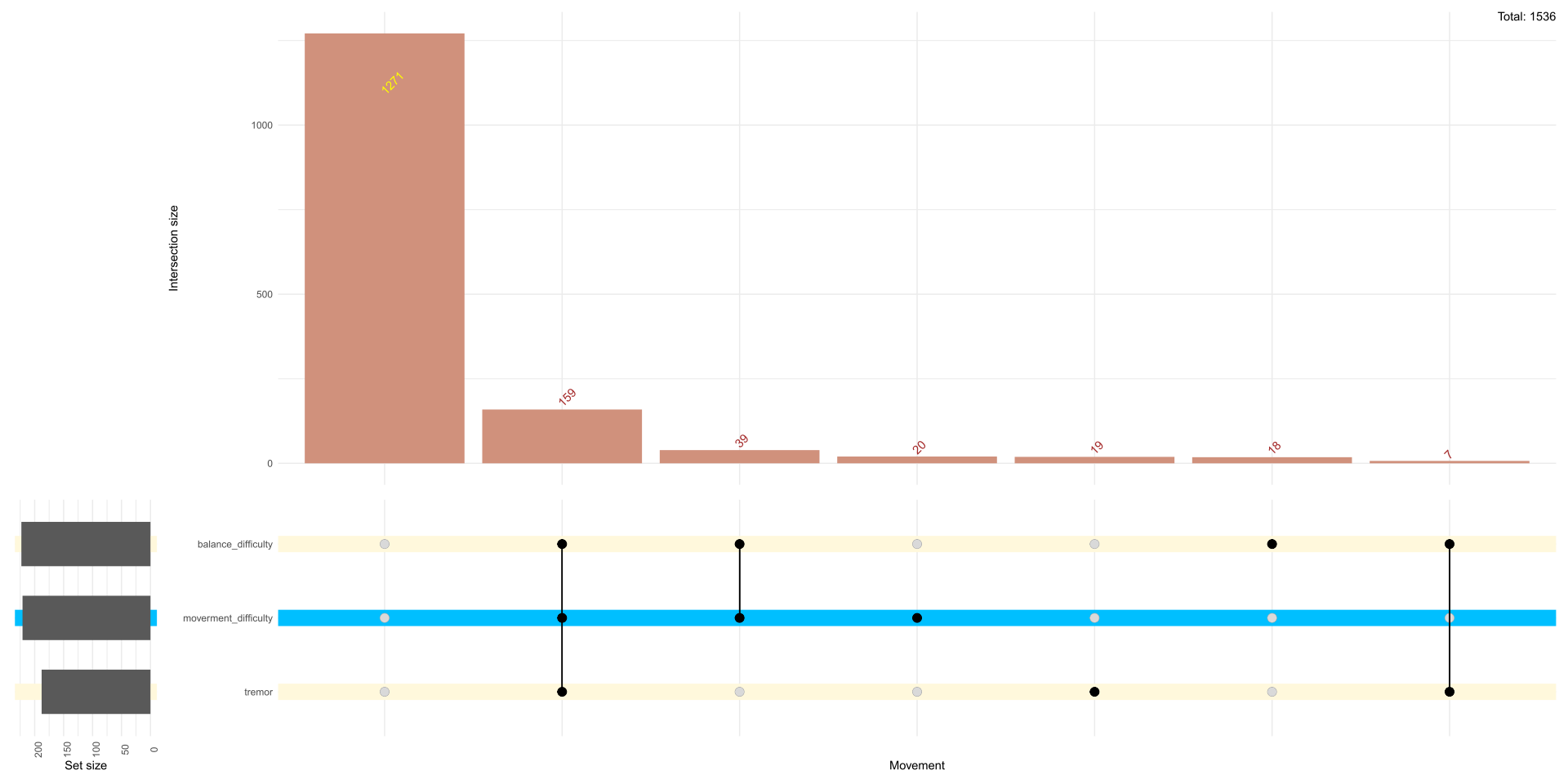

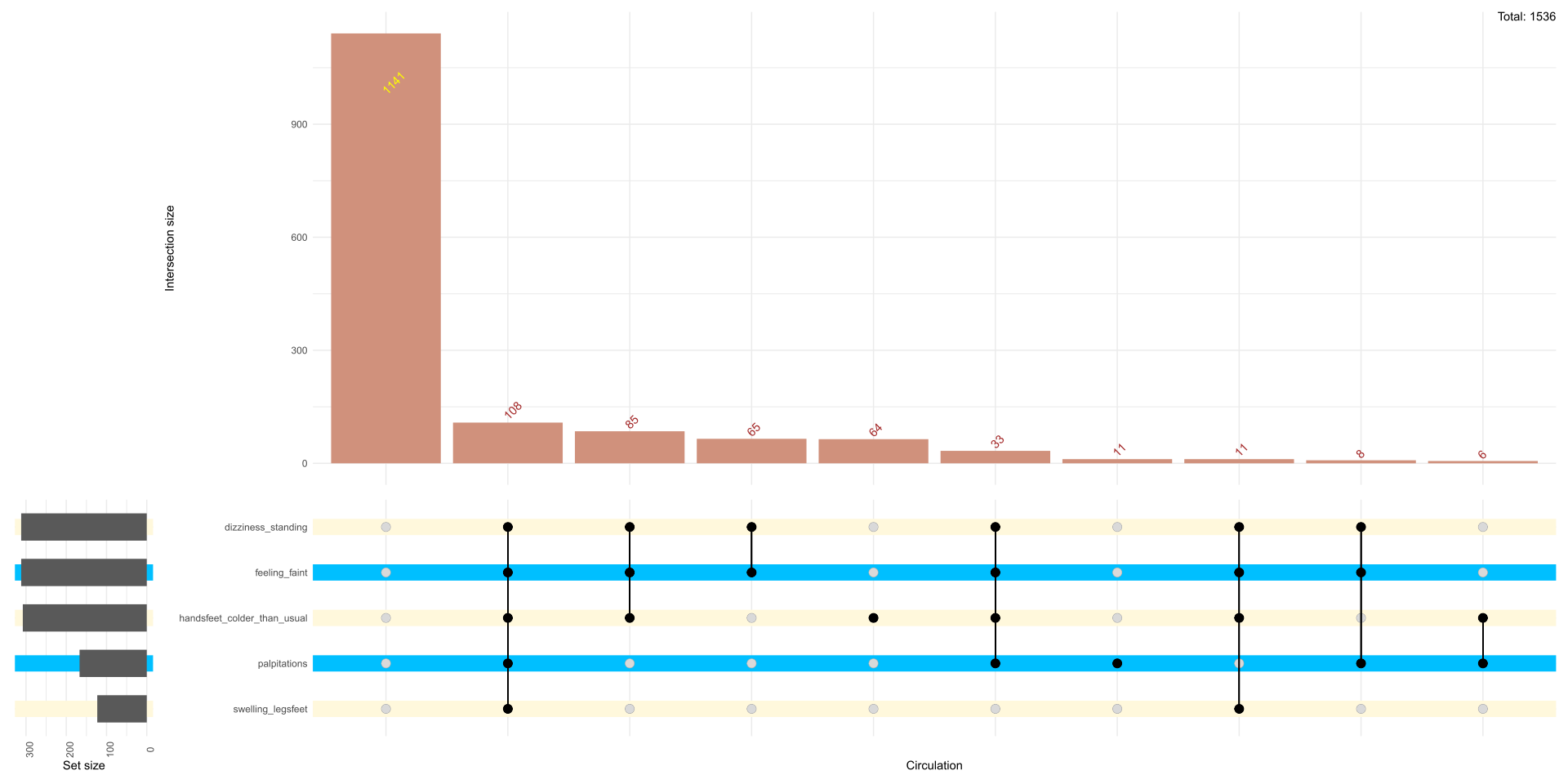

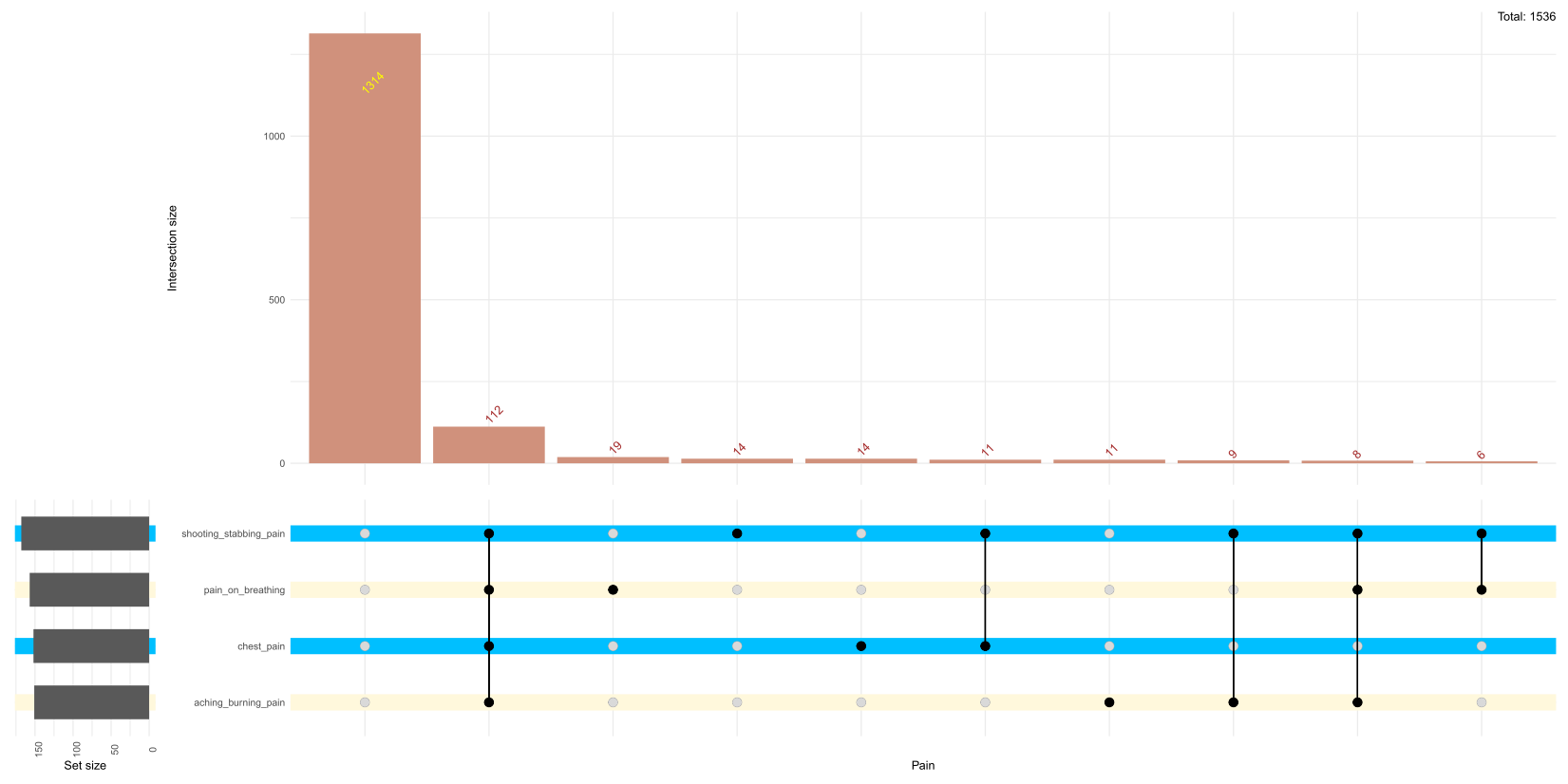

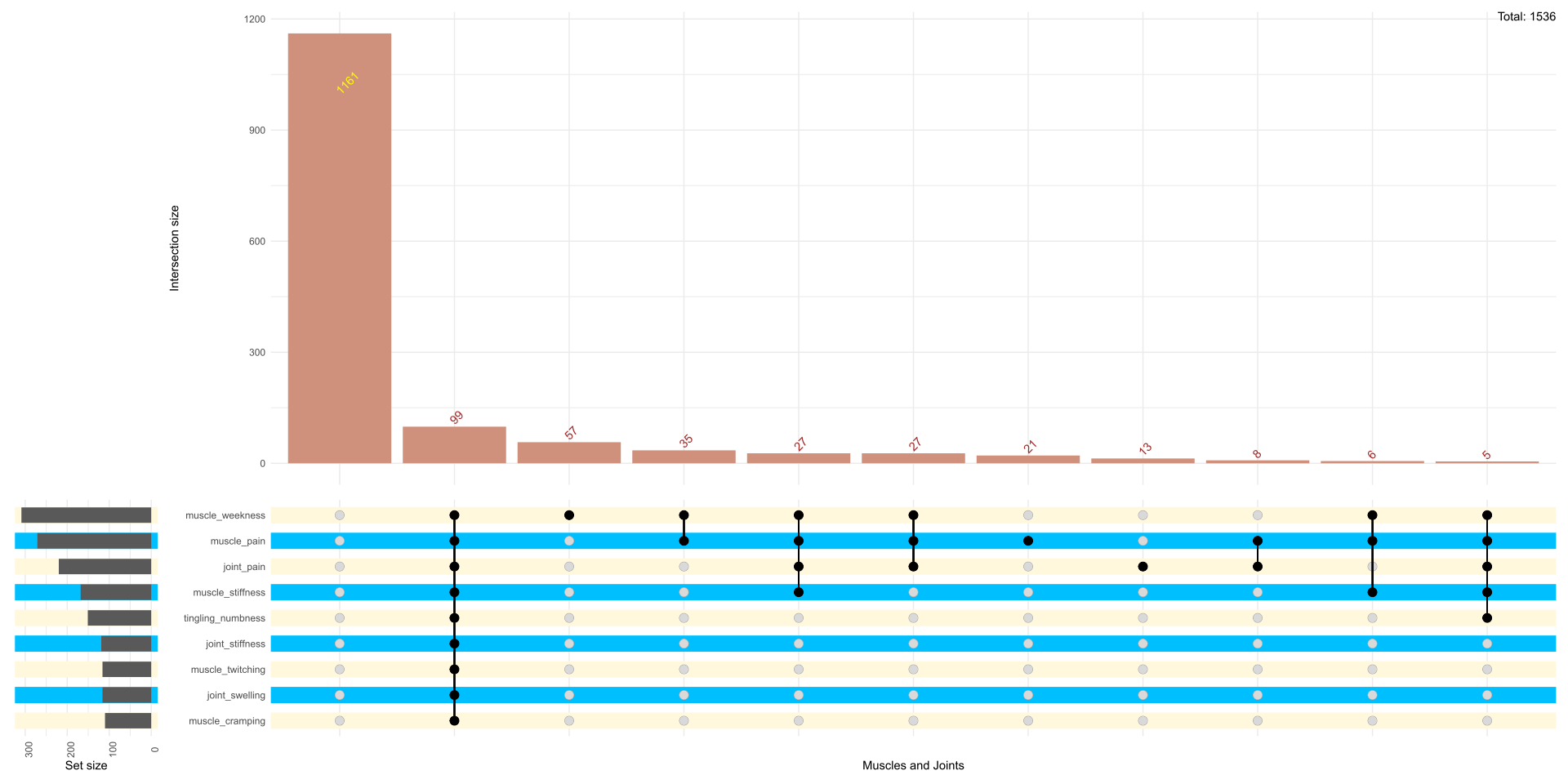

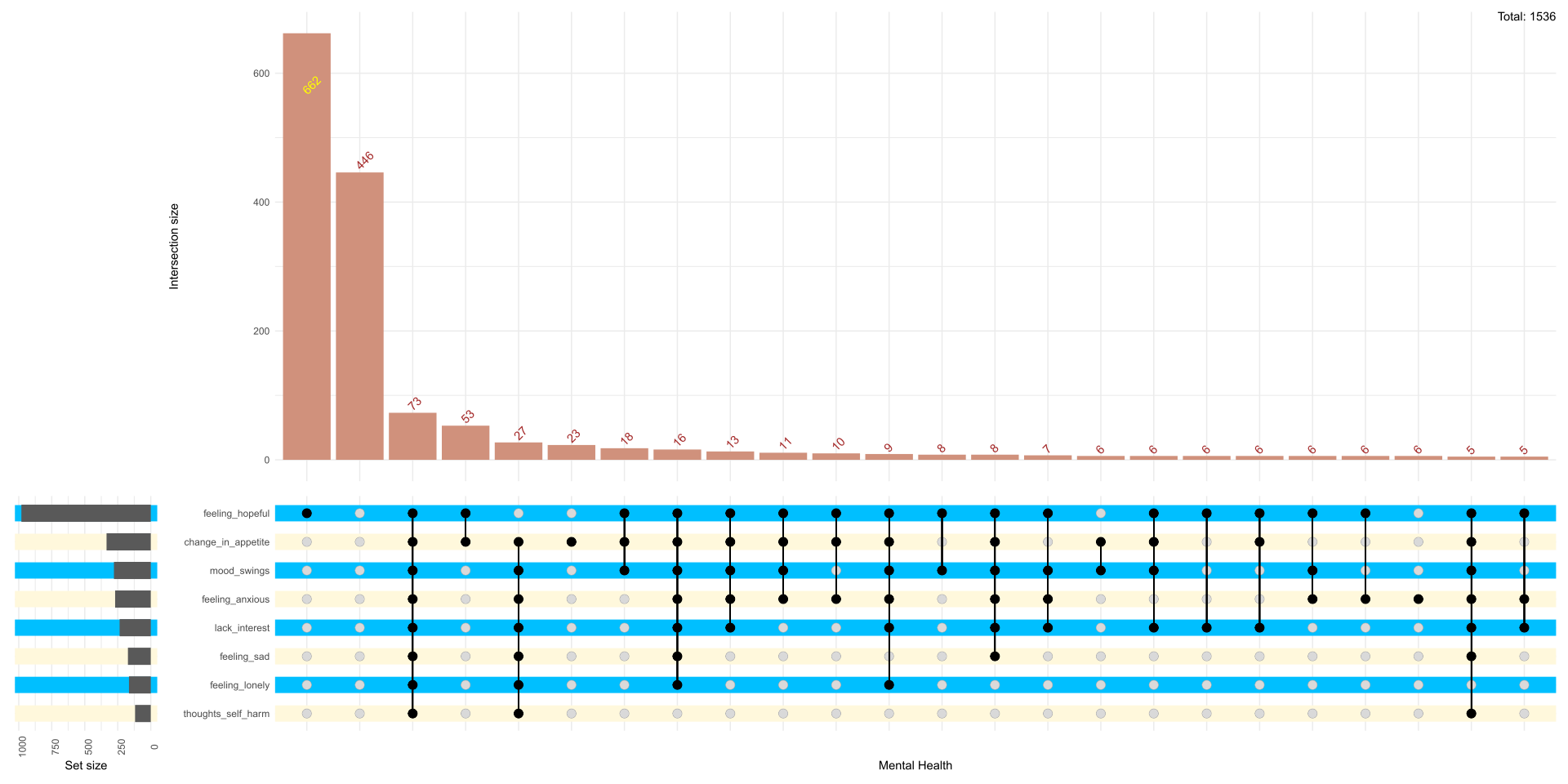

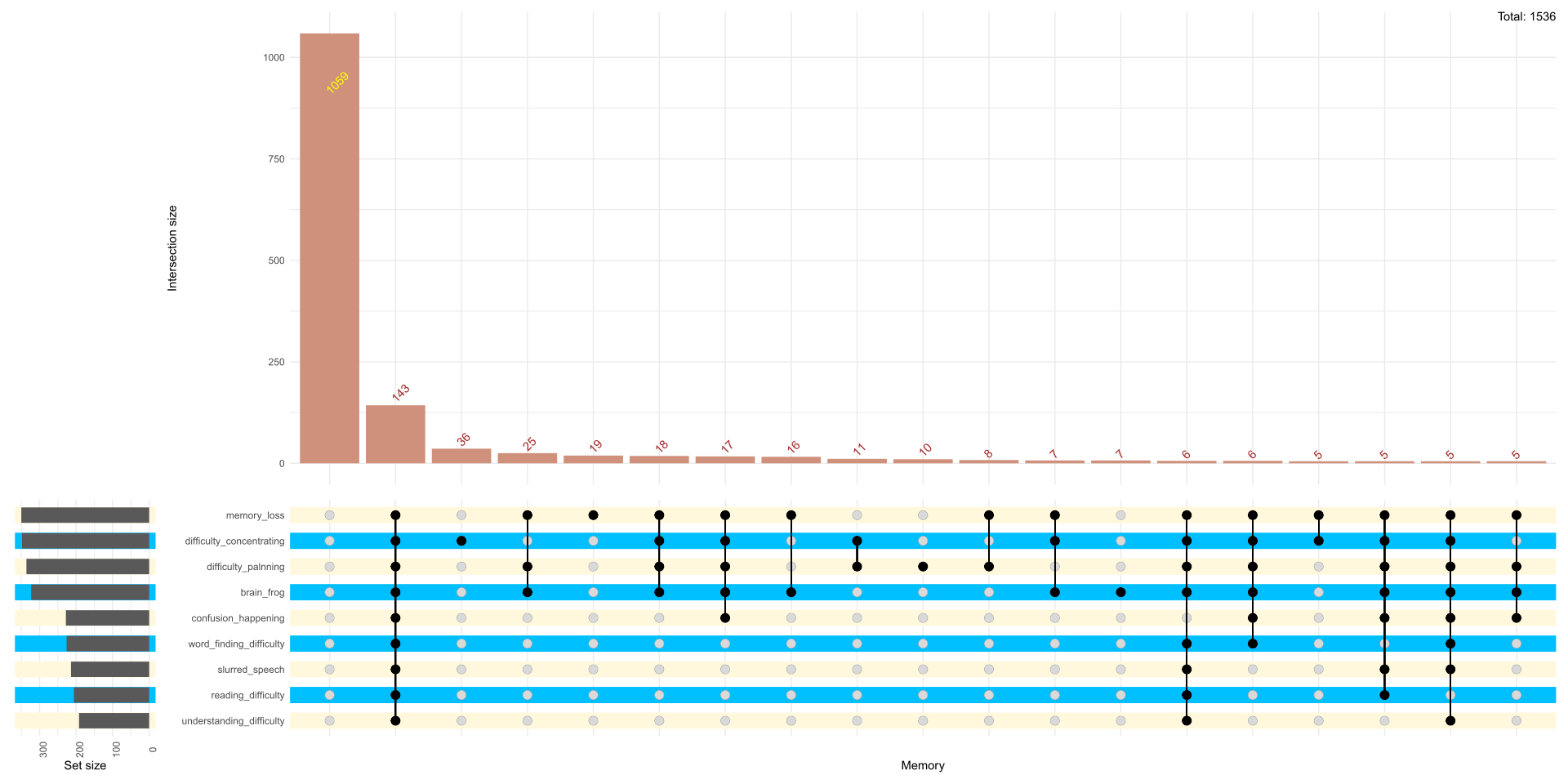


**Supplementary Figure 6.** Distribution of symptom combinations within each domain in the third survey.


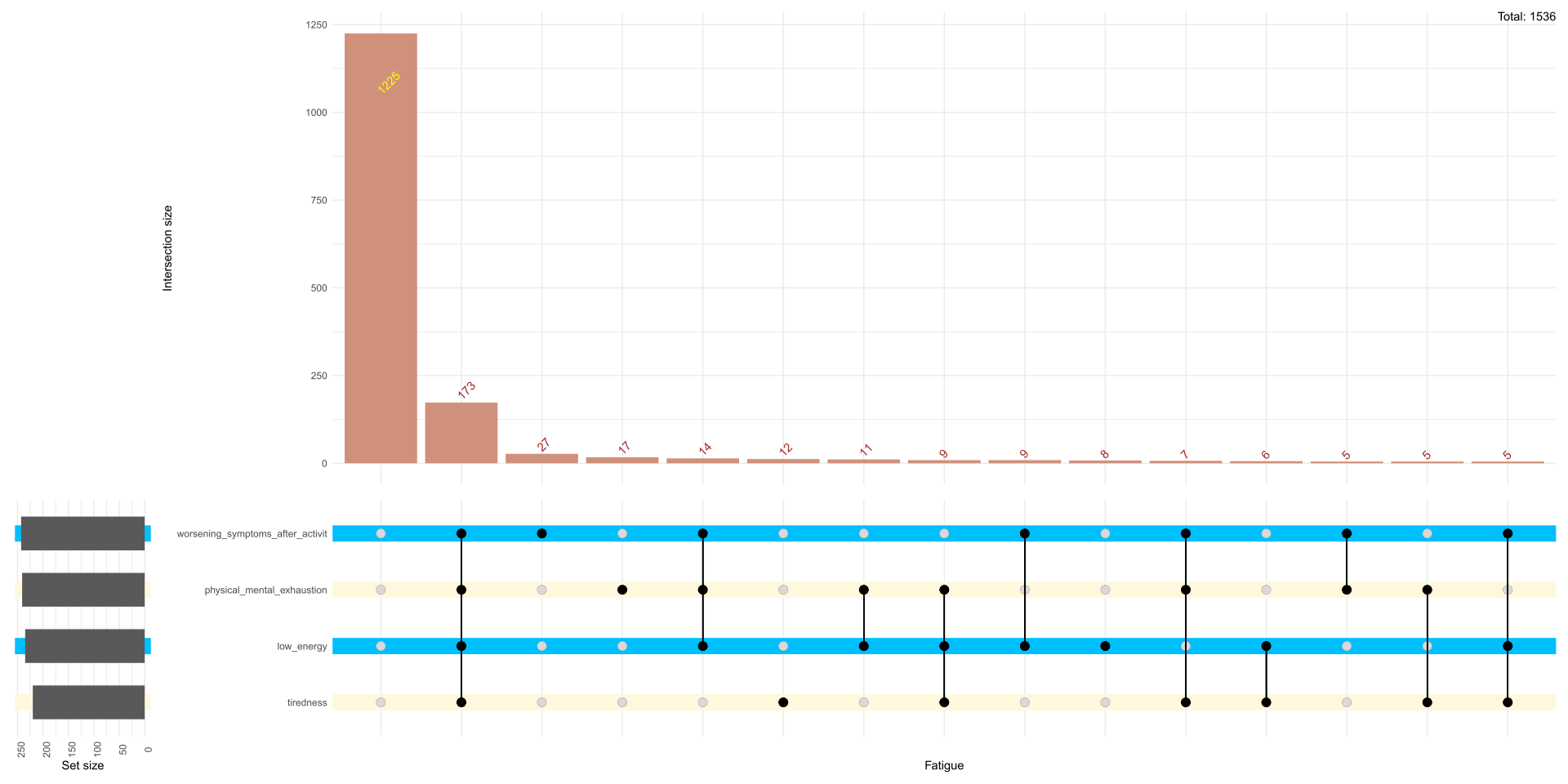


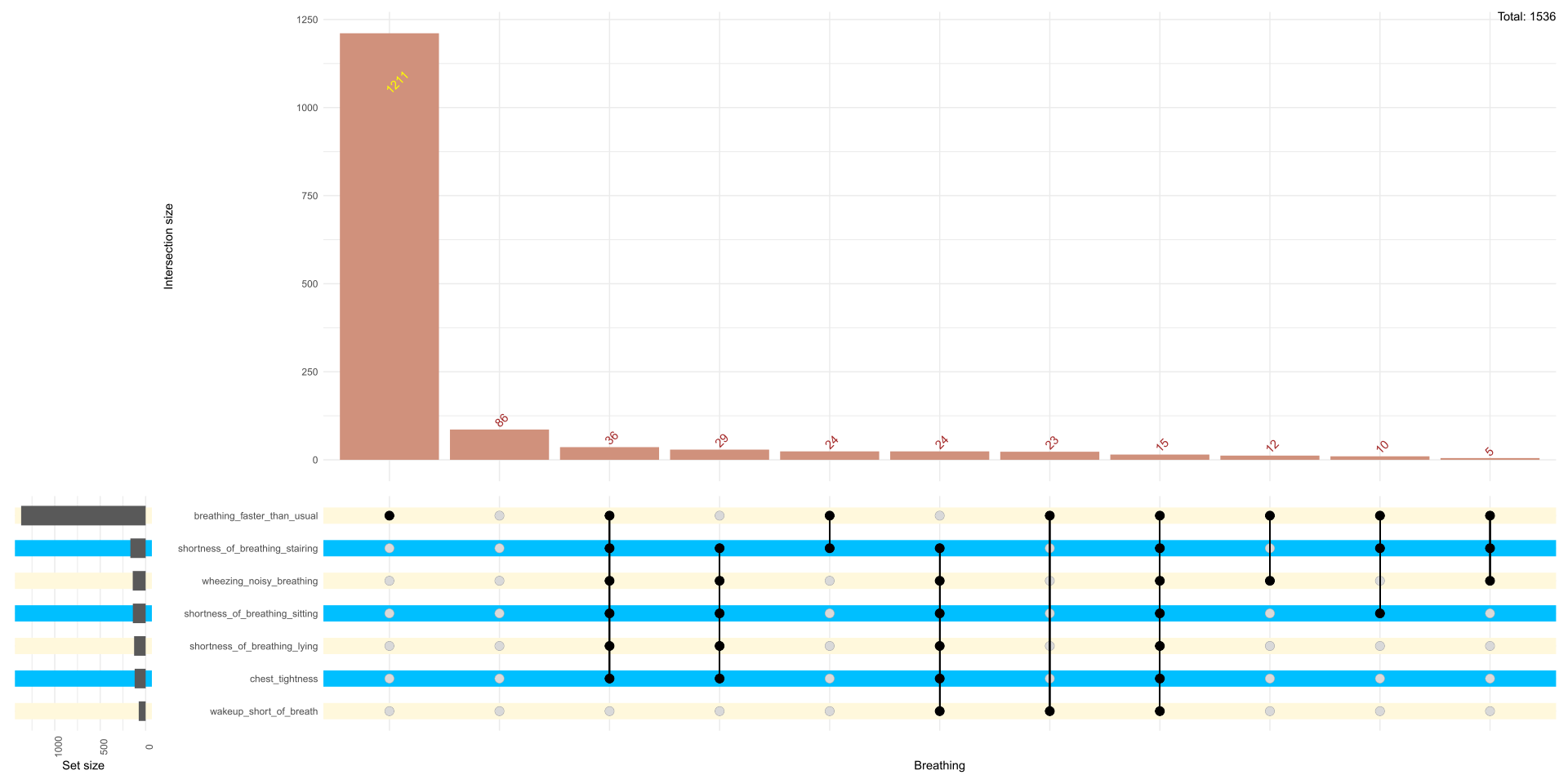

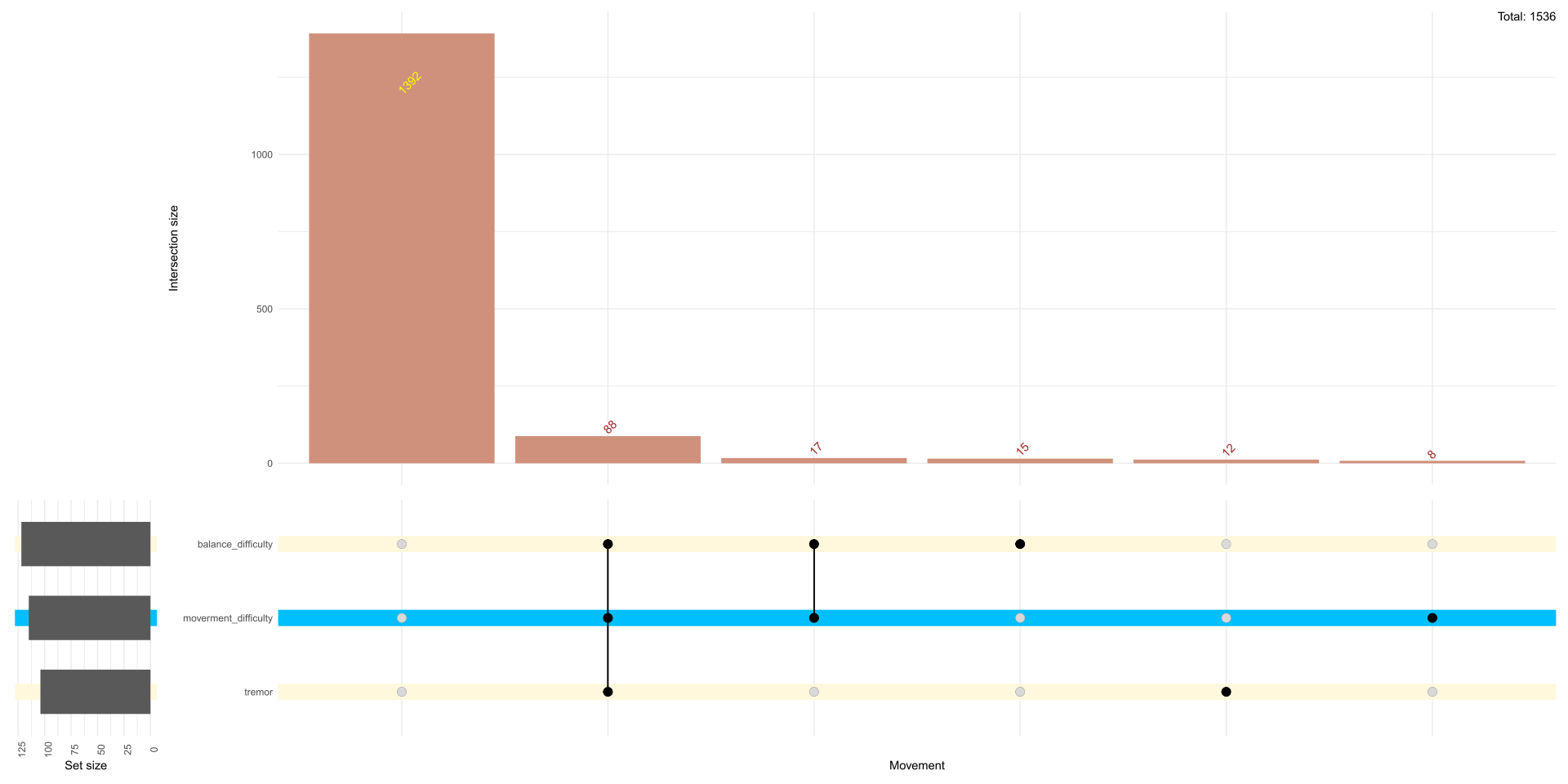

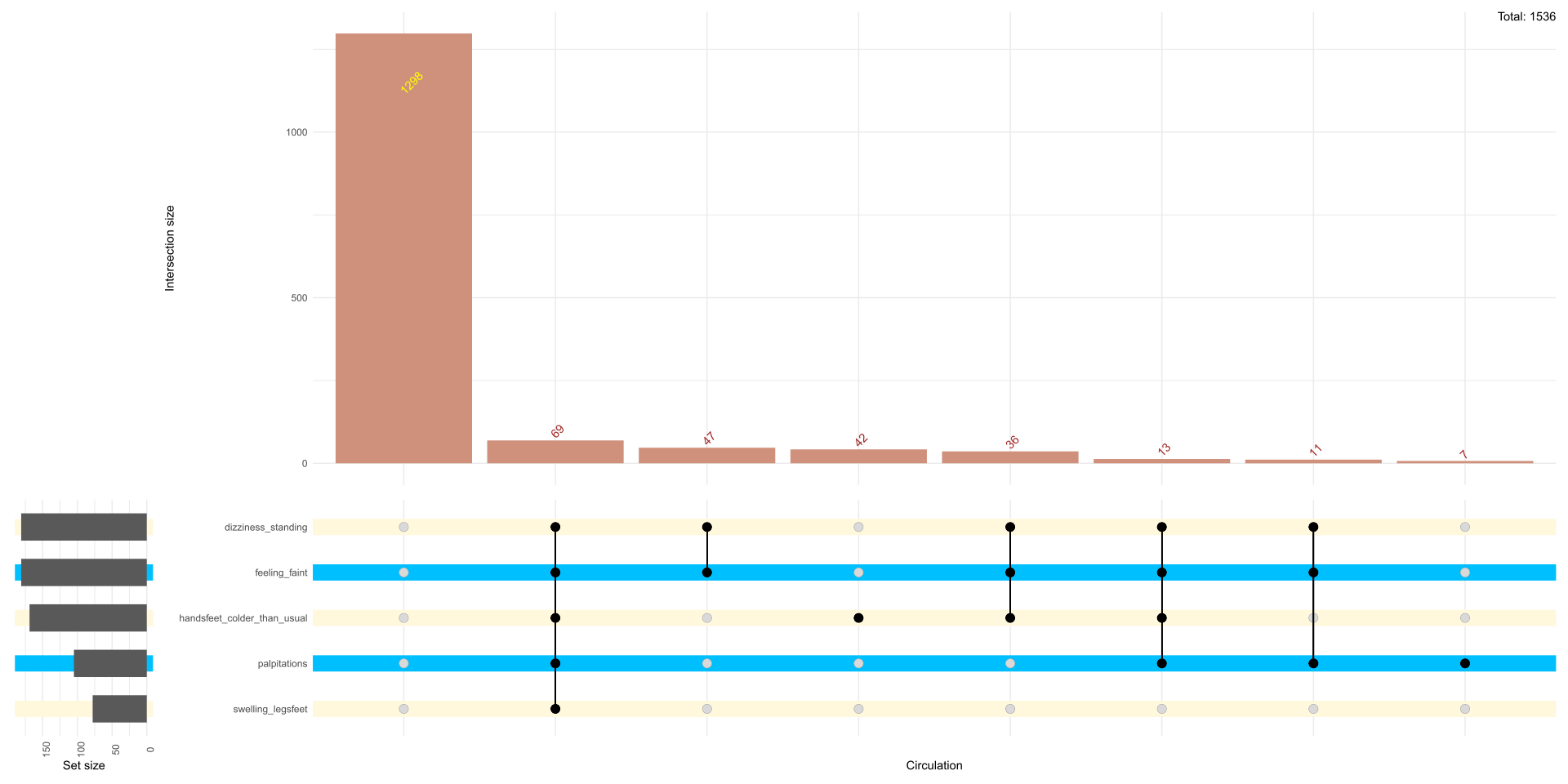

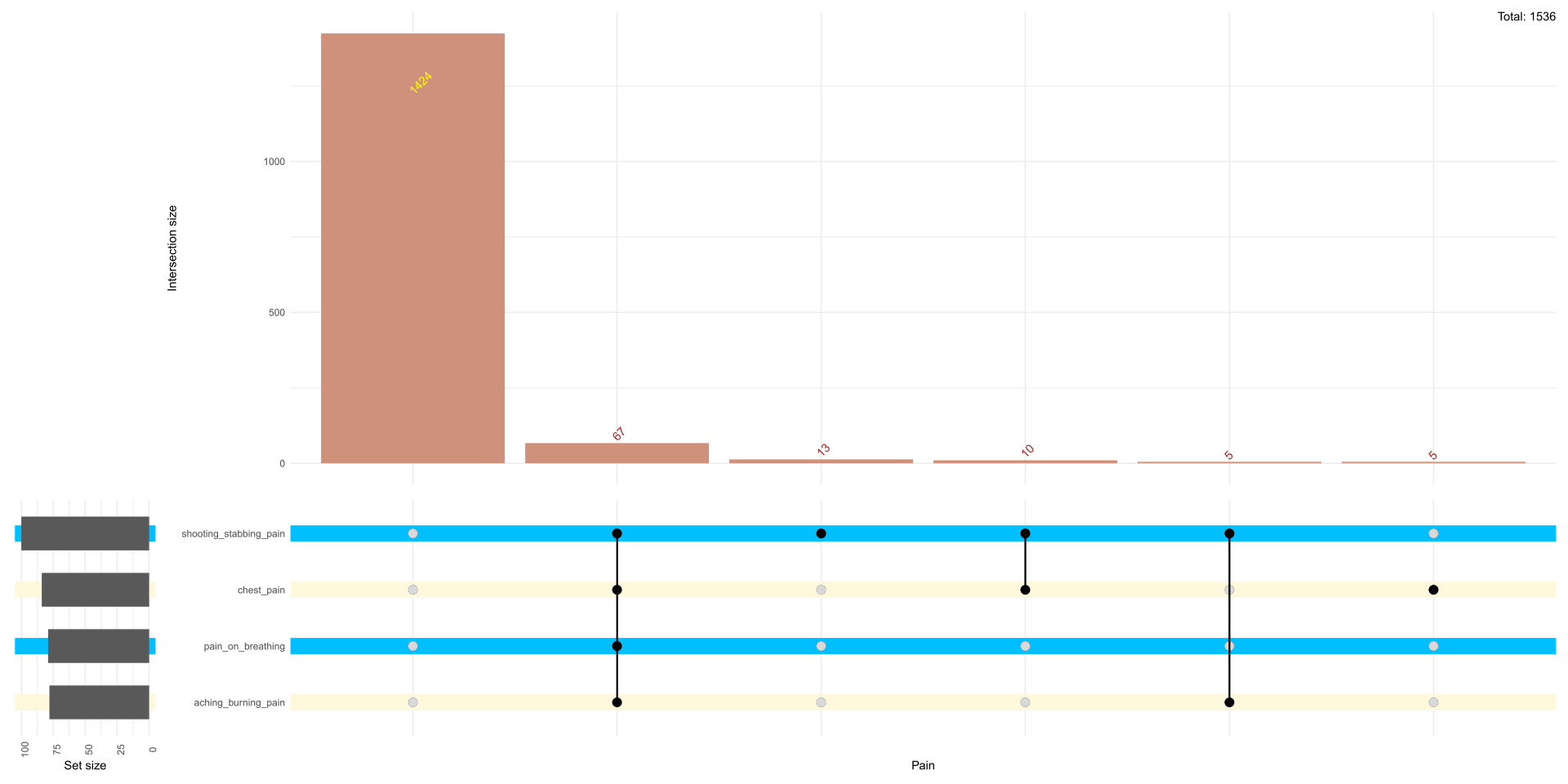

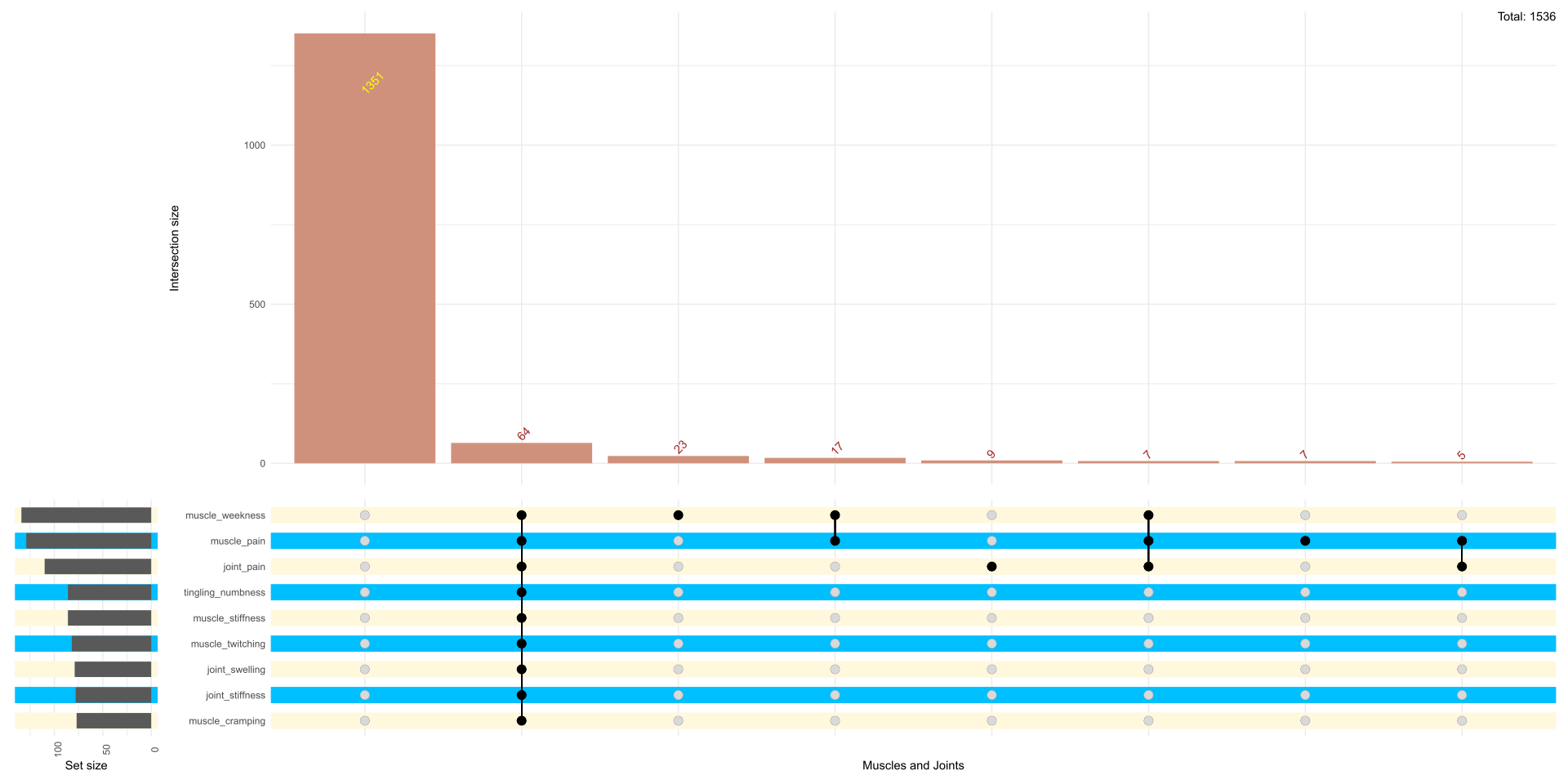

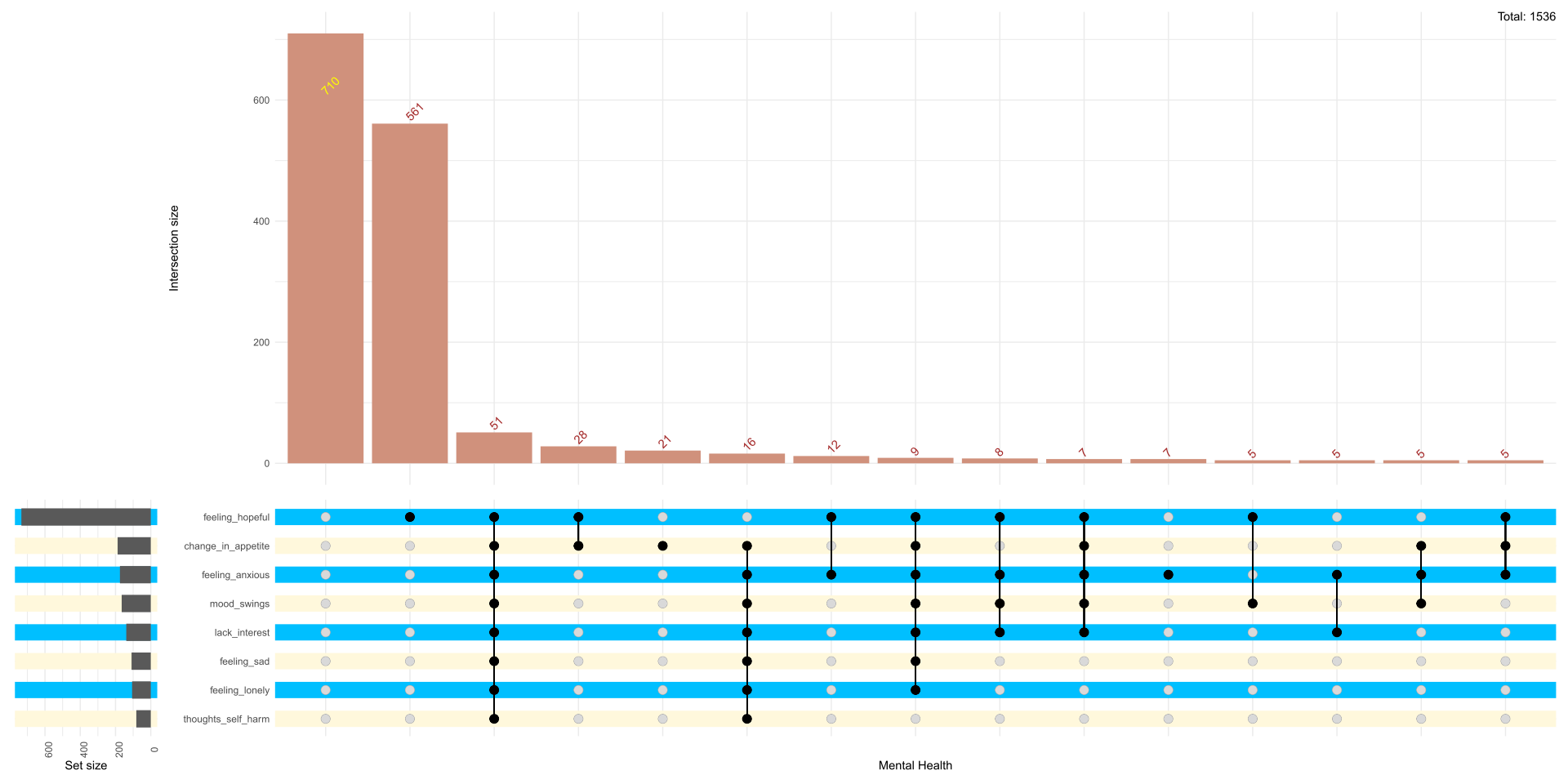

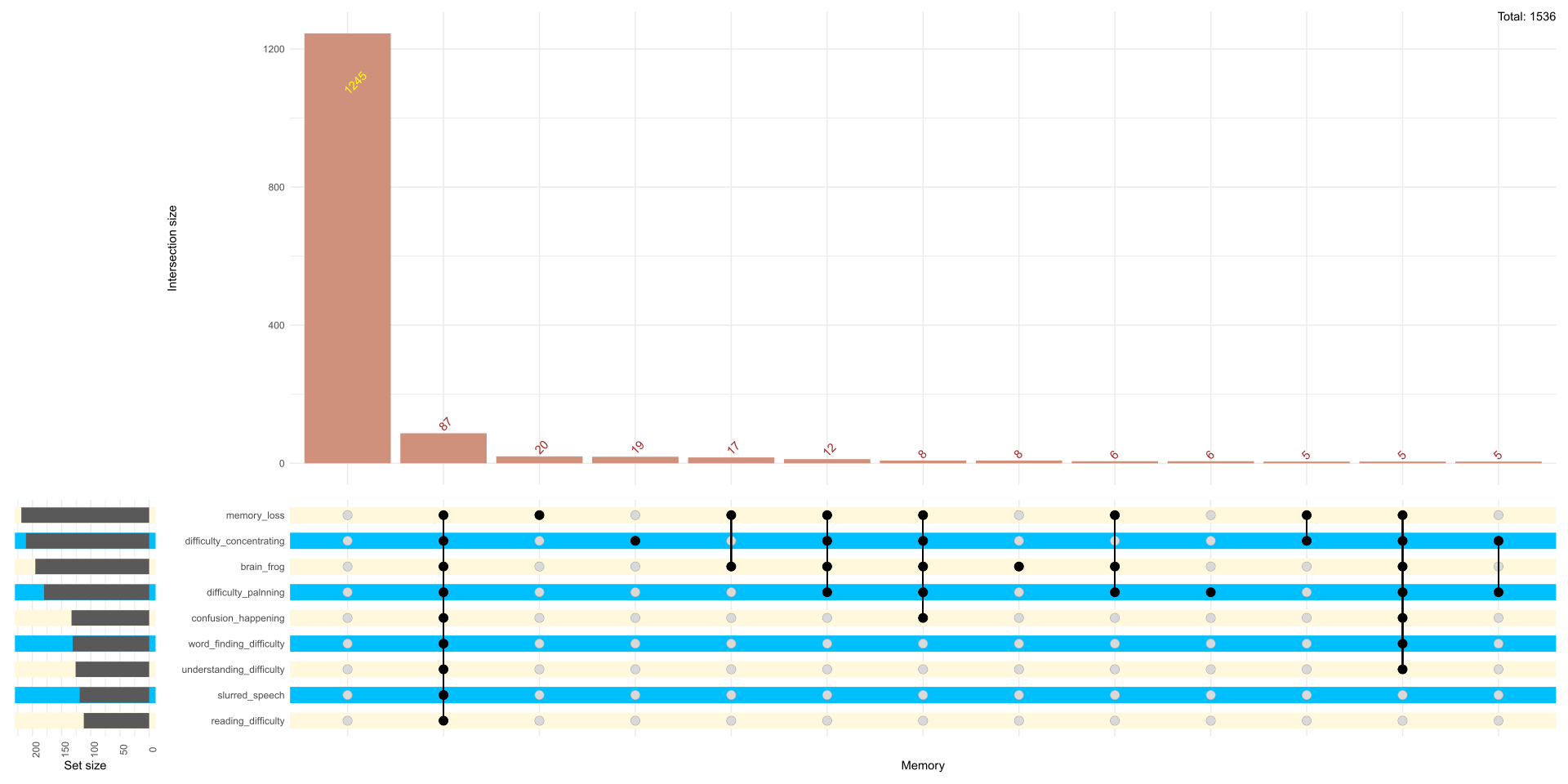


**Supplementary Figure 7.** Distribution of symptom combinations within each domain in the fourth survey.


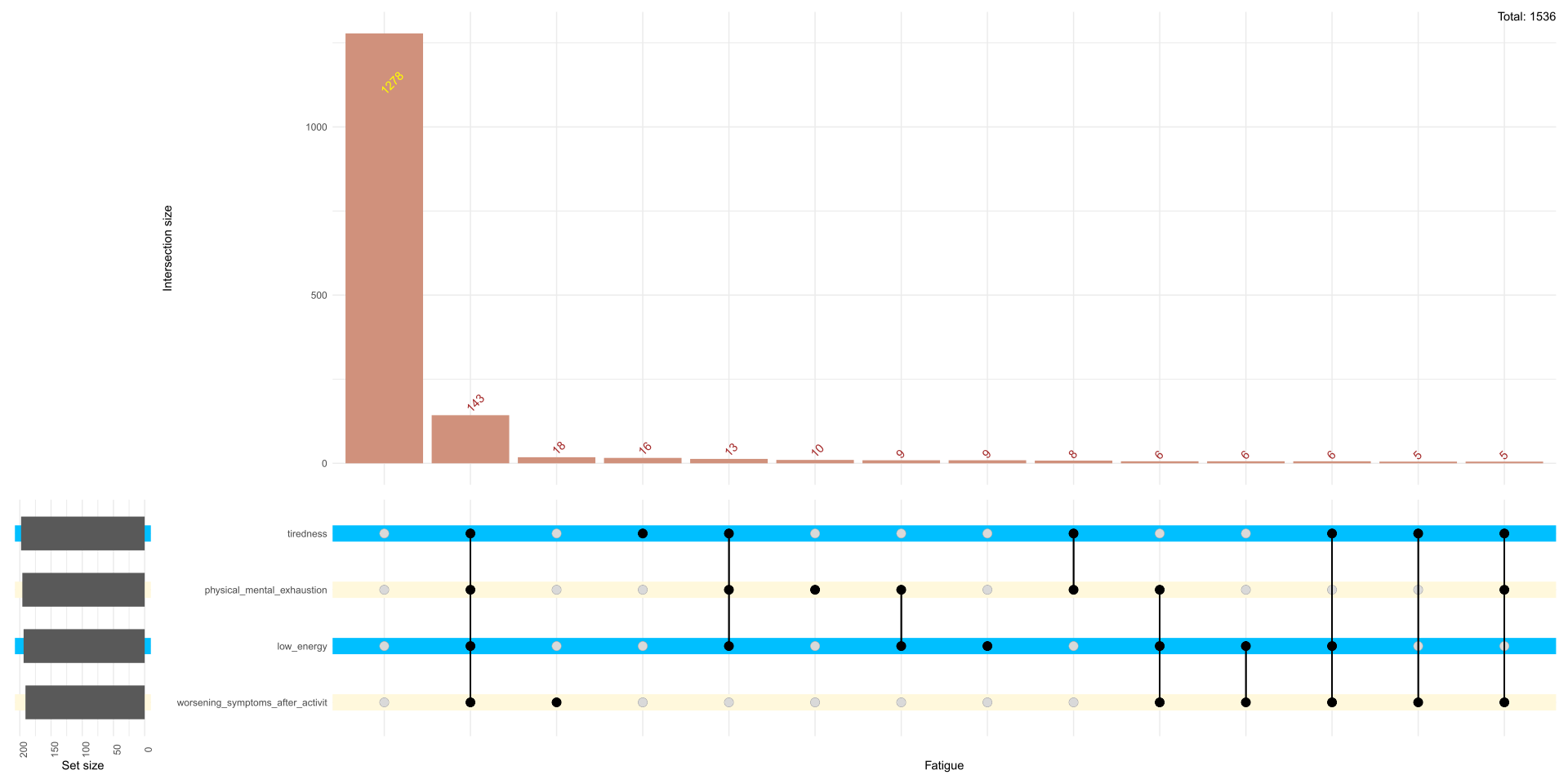


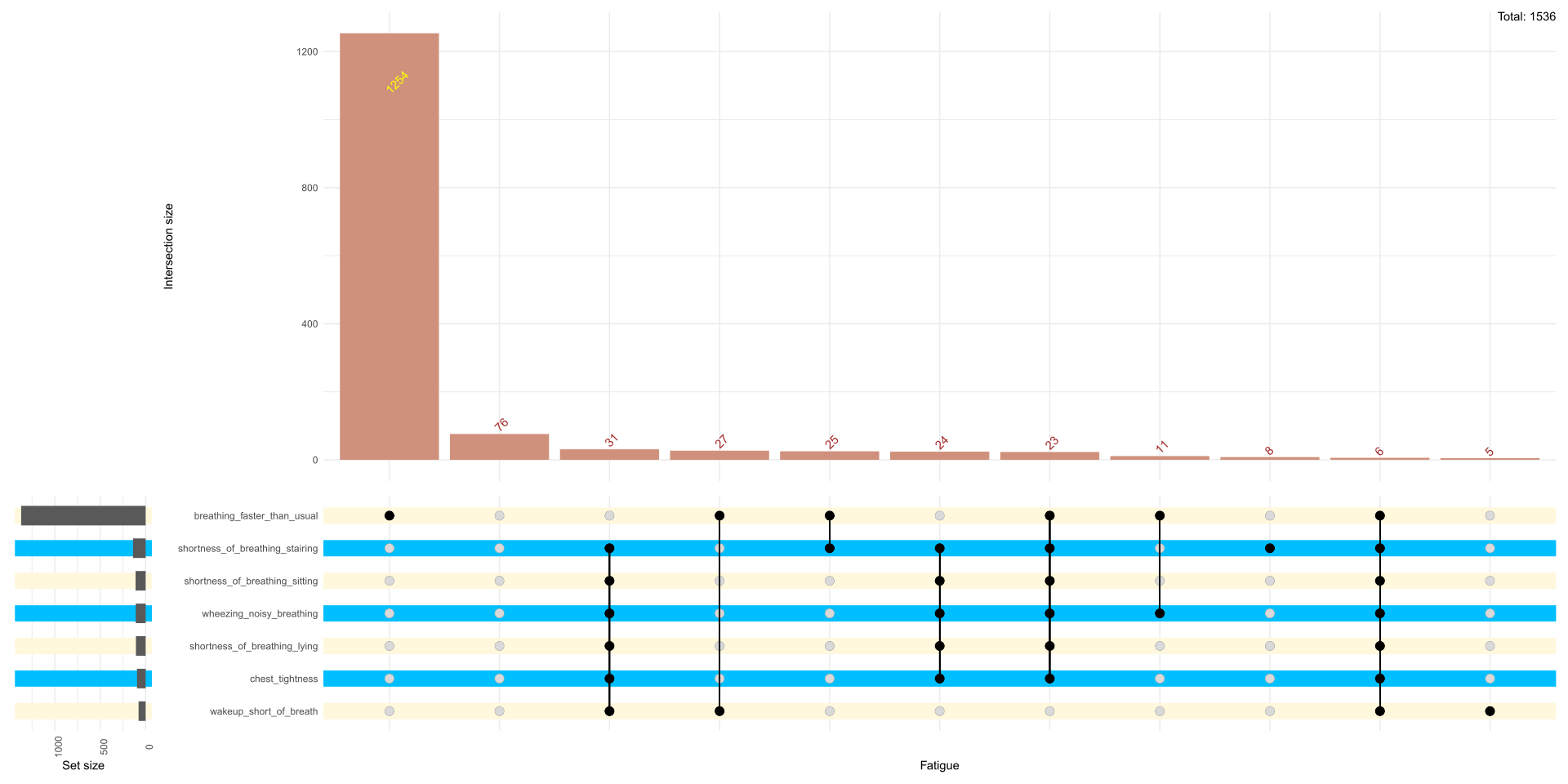

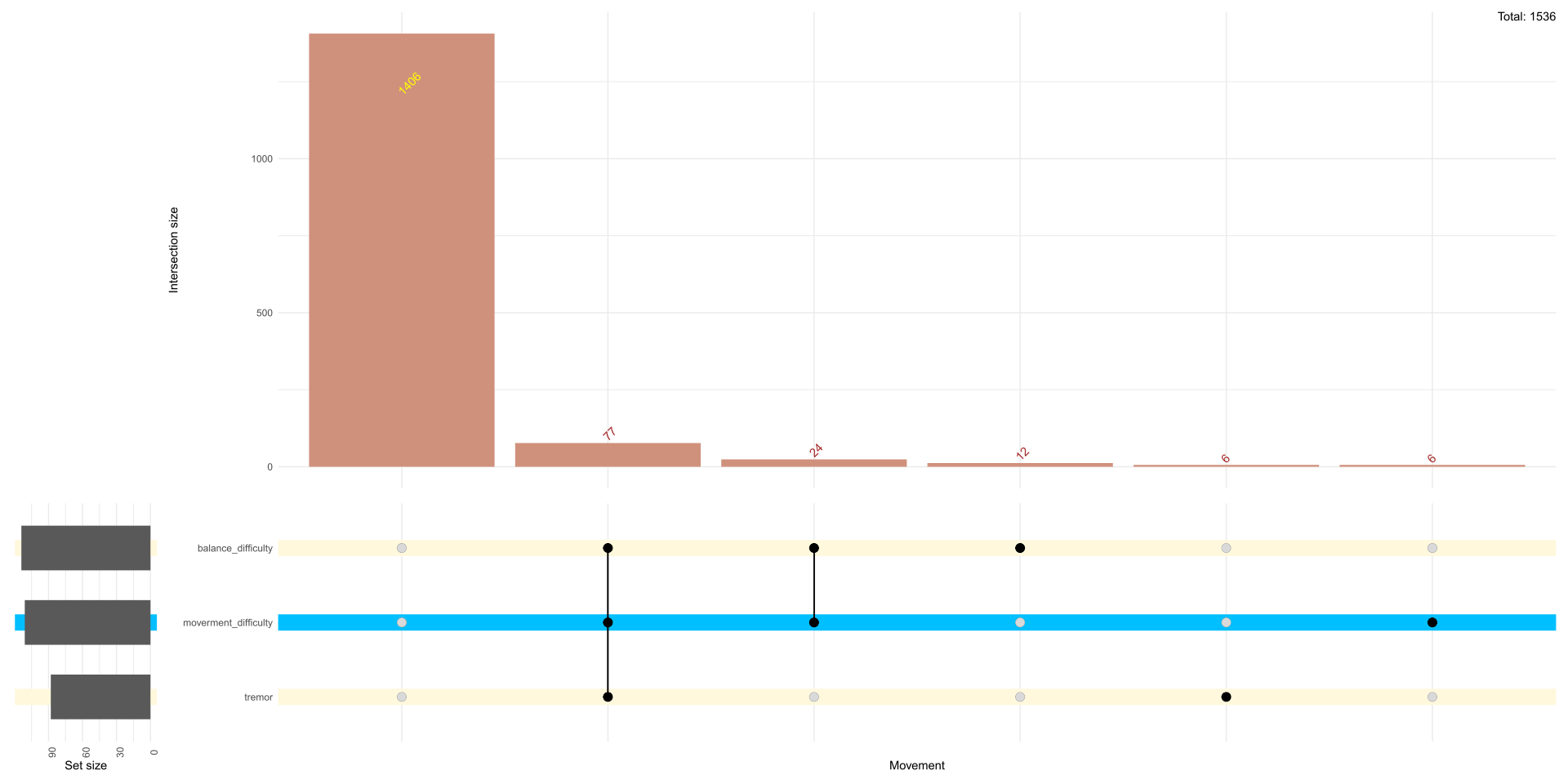

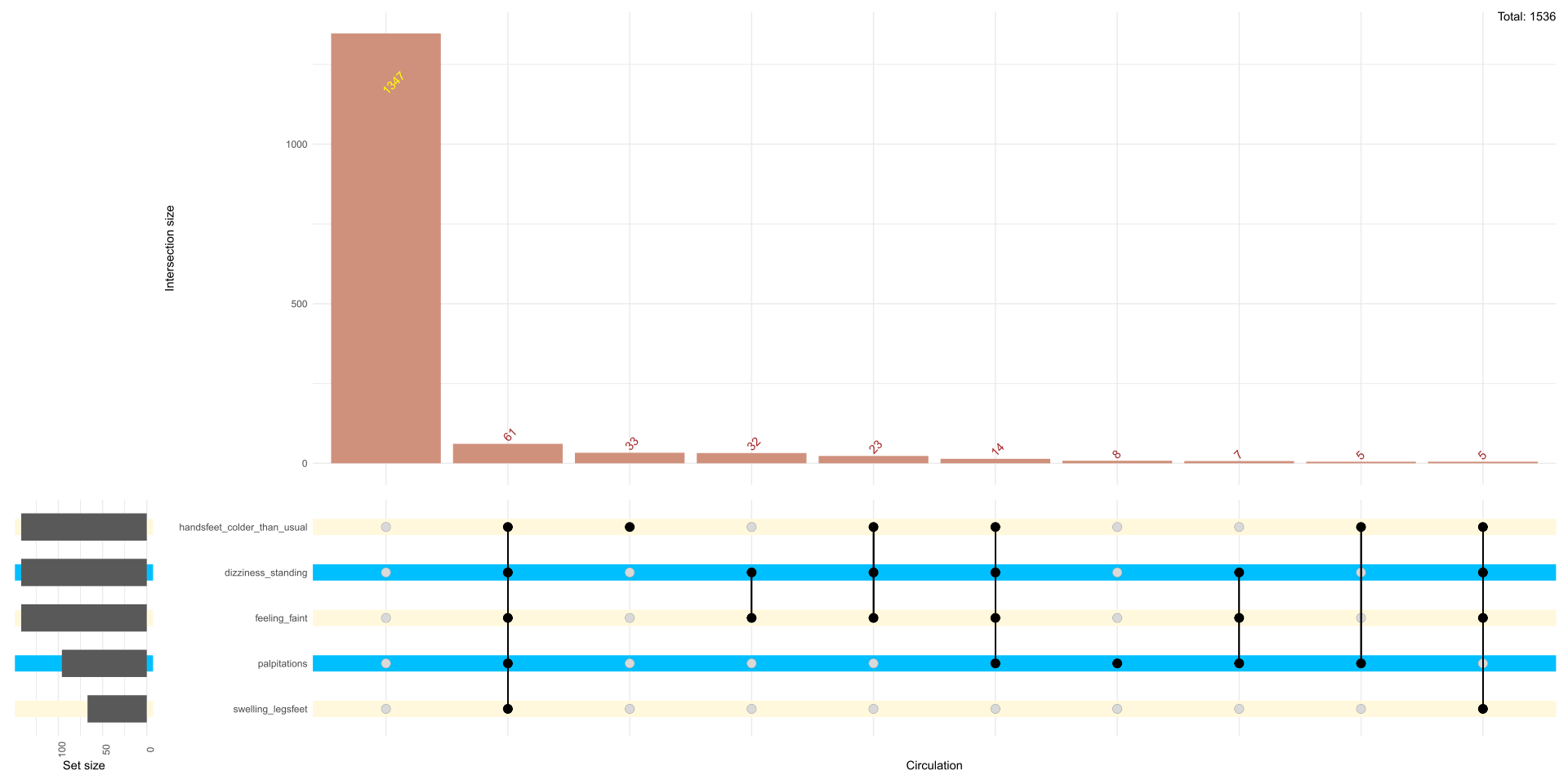

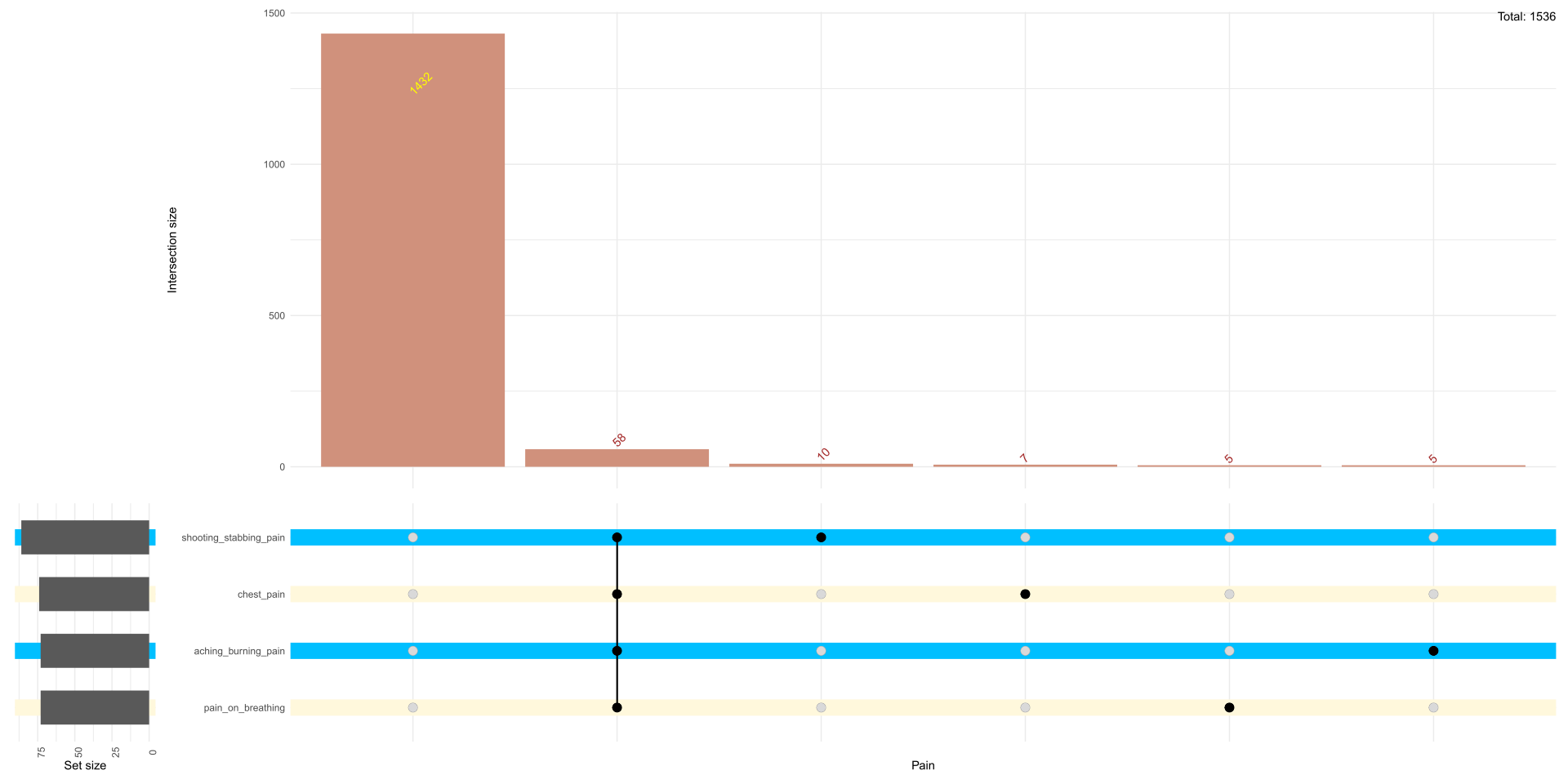

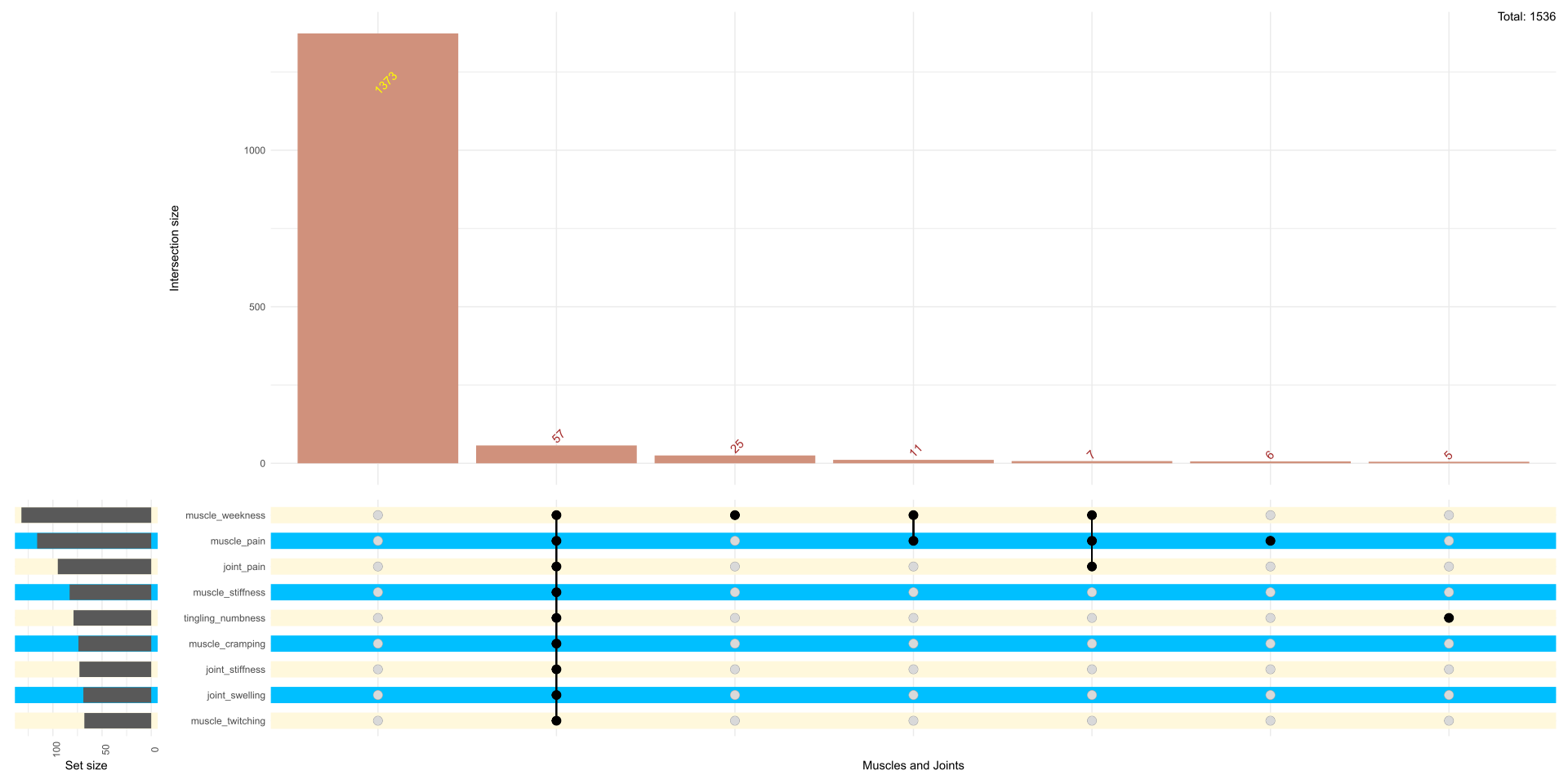

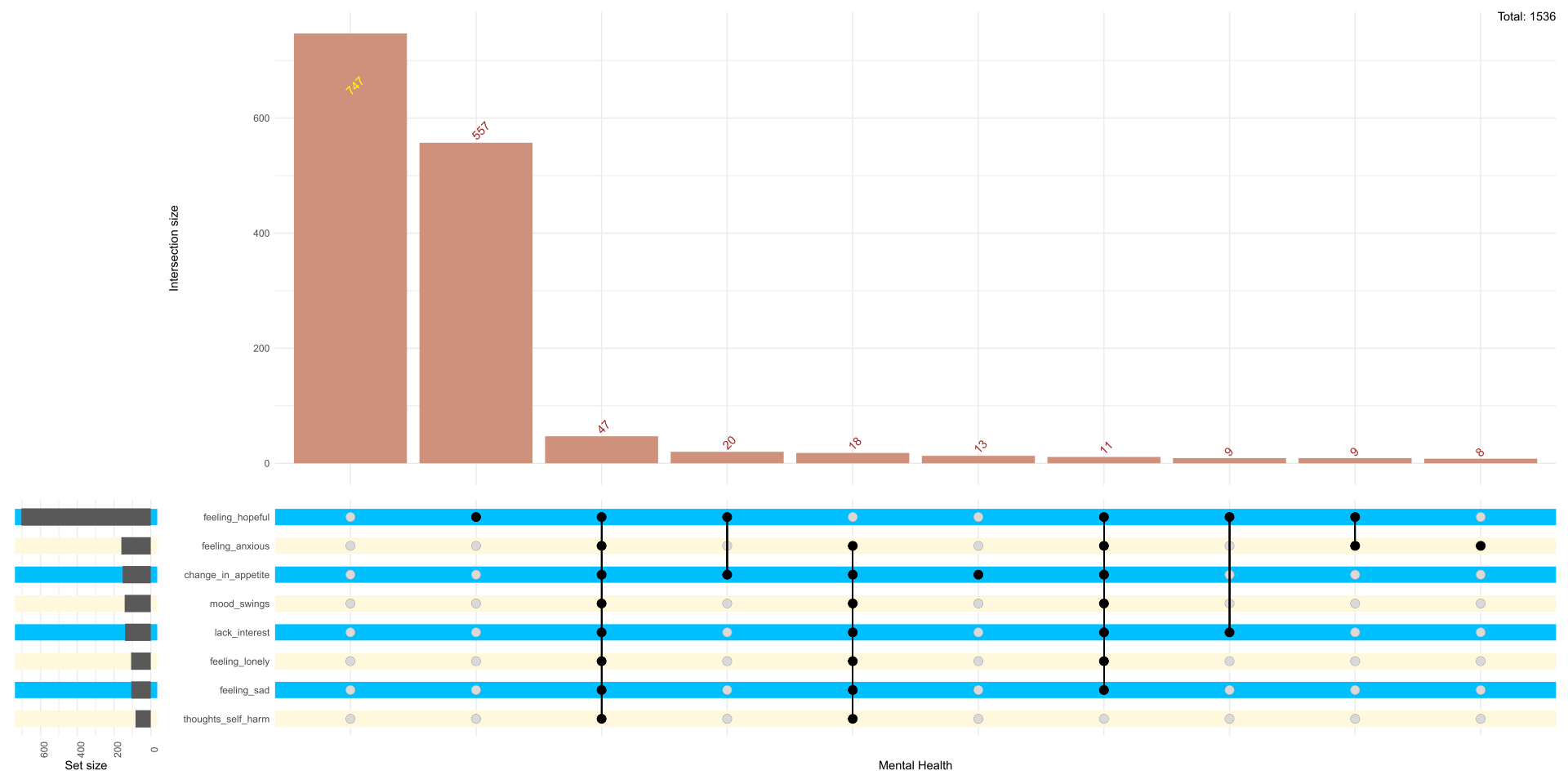

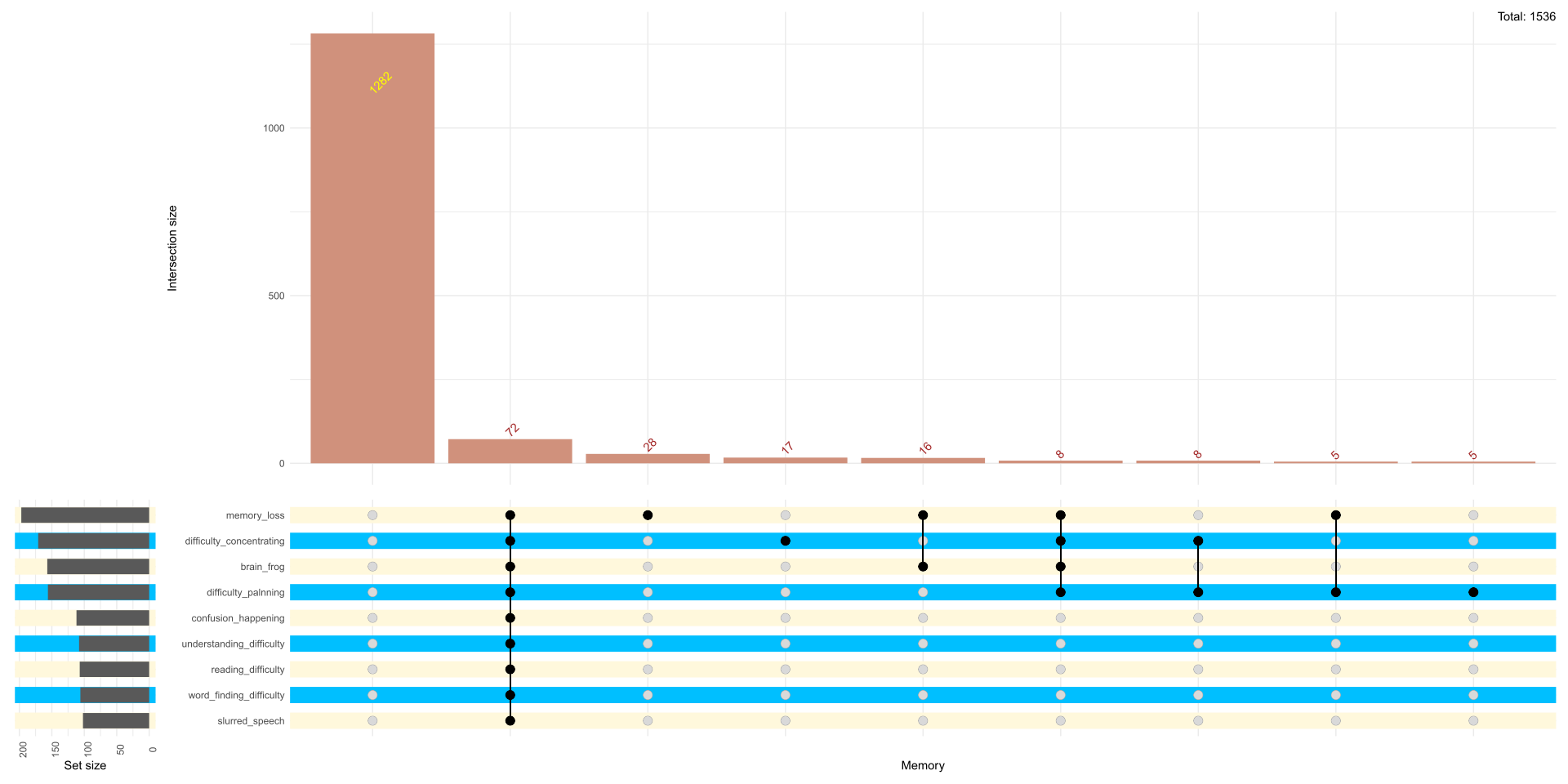

Supplement: Supplementary file 1 [file Supplementary_file_1.docx]
